# Mental health problems among female sex workers in low- and middle-income countries: A systematic review and meta-analysis

**DOI:** 10.1371/journal.pmed.1003297

**Published:** 2020-09-15

**Authors:** Tara S. Beattie, Boryana Smilenova, Shari Krishnaratne, April Mazzuca

**Affiliations:** 1 Department of Global Health and Development, The London School of Hygiene & Tropical Medicine, London, United Kingdom; 2 King’s Health Partners, Guy’s Hospital, London, United Kingdom; 3 School of Population and Public Health, University of British Columbia, Canada; Harvard Medical School, UNITED STATES

## Abstract

**Background:**

The psychological health of female sex workers (FSWs) has emerged as a major public health concern in many low- and middle-income countries (LMICs). Key risk factors include poverty, low education, violence, alcohol and drug use, human immunodeficiency virus (HIV), and stigma and discrimination. This systematic review and meta-analysis aimed to quantify the prevalence of mental health problems among FSWs in LMICs, and to examine associations with common risk factors.

**Method and findings:**

The review protocol was registered with PROSPERO, number CRD42016049179. We searched 6 electronic databases for peer-reviewed, quantitative studies from inception to 26 April 2020. Study quality was assessed with the Centre for Evidence-Based Management (CEBM) Critical Appraisal Tool. Pooled prevalence estimates were calculated for depression, anxiety, post-traumatic stress disorder (PTSD), and suicidal behaviour. Meta-analyses examined associations between these disorders and violence, alcohol/drug use, condom use, and HIV/sexually transmitted infection (STI). A total of 1,046 studies were identified, and 68 papers reporting on 56 unique studies were eligible for inclusion. These were geographically diverse (26 countries), representing all LMIC regions, and included 24,940 participants. All studies were cross-sectional and used a range of measurement tools; none reported a mental health intervention. Of the 56 studies, 14 scored as strong quality, 34 scored as moderate, and 8 scored as weak. The average age of participants was 28.9 years (age range: 11–64 years), with just under half (46%) having up to primary education or less. The pooled prevalence rates for mental disorders among FSWs in LMICs were as follows: depression 41.8% (95% CI 35.8%–48.0%), anxiety 21.0% (95% CI: 4.8%–58.4%), PTSD 19.7% (95% CI 3.2%–64.6%), psychological distress 40.8% (95% CI 20.7%–64.4%), recent suicide ideation 22.8% (95% CI 13.2%–36.5%), and recent suicide attempt 6.3% (95% CI 3.4%–11.4%). Meta-analyses found significant associations between violence experience and depression, violence experience and recent suicidal behaviour, alcohol use and recent suicidal behaviour, illicit drug use and depression, depression and inconsistent condom use with clients, and depression and HIV infection. Key study limitations include a paucity of longitudinal studies (necessary to assess causality), non-random sampling of participants by many studies, and the use of different measurement tools and cut-off scores to measure mental health problems and other common risk factors.

**Conclusions:**

In this study, we found that mental health problems are highly prevalent among FSWs in LMICs and are strongly associated with common risk factors. Study findings support the concept of overlapping vulnerabilities and highlight the urgent need for interventions designed to improve the mental health and well-being of FSWs.

## Abstract 2

Tara Beattie and co-workers assess the evidence on mental ill-health in female sex workers in low- and middle-income countries.

## Author summary

**Why was this study done?:**

- Understanding the prevalence of mental health problems, and key risk factors associated with poor mental health, is essential for building evidence-based prevention.
- To our knowledge, there has been no previous quantitative synthesis of the literature examining the prevalence of mental health problems among female sex workers (FSWs) in low- and middle-income countries (LMICs).
- This evidence is critical to inform evidence-based policymaking and intervention design and is timely given the growing interest in mental health globally.

**What did the researchers do and find?:**

- We undertook a quantitative systematic review to estimate the prevalence of mental health problems and to estimate the magnitude of associations between mental health problems and sex workers’ experience of violence, harmful alcohol and drug use, condom use, and human immunodeficiency virus (HIV) and sexually transmitted infections (STIs).
- Meta-analyses across 31 studies with 18,524 FSWs in 17 LMICs suggest that mental health problems—including depression, anxiety, post-traumatic stress disorder (PTSD), and suicidal thoughts and attempts—are highly prevalent among FSWs.
- Sex workers with a mental health problem were more likely to have experienced violence, to report harmful alcohol or drug use, to report inconsistent condom use with clients (but not regular partners), and to be HIV positive compared with sex workers who did not have a mental health problem (18 studies with 14,115 FSWs).

**What do these findings mean?:**

- The quantitative evidence clearly demonstrates that mental health problems are an important burden of disease experienced by many FSWs in LMICs, with levels substantially greater than those experienced by women in the general population.
- As no study described a mental health intervention, evidence-based mental health interventions for FSWs are urgently required to address the current treatment gap. The prevention and treatment of key risk factors such as violence and harmful alcohol and drug use would also help improve the mental health of FSWs.
- Longitudinal research is needed in order to unpack pathways of causation. Future studies should use validated tools and consistent cut-offs and timeframes to enable more rigorous comparisons between studies.

## Introduction

Mental health problems are a significant cause of the global burden of disease. In 2010, mental, neurological, and substance use disorders were the leading cause of years lived with disability globally [1]. Worldwide, an estimated 300 million people are affected by depression, and 272 million people by anxiety, with women at higher risk compared with men [2,3]. The treatment gap for common conditions exceeds more than 90% in low-income countries [4]. Left untreated, mental disorders prevent people from reaching their full potential, impair human capital, and are associated with premature mortality from suicide and other illnesses [5]. Suicide is a health outcome strongly associated with mental, neurological, and substance use disorders. Nearly 800,000 people are estimated to die due to suicide each year, with 79% of global suicides occurring in low- and middle-income countries (LMICs) [6]. A range of social determinants affect the risk and outcome of mental disorders. These include demographic factors (such as age, gender, and ethnicity), socioeconomic factors (such as low income, unemployment, and low education), neighbourhood factors (such as inadequate housing and neighbourhood violence), and social change associated with changes in income and urbanisation [1].

Sex work—defined by The Joint United Nations Programme on HIV/AIDS (UNAIDS) as the receipt of money or goods in exchange for sexual services, either regularly or occasionally [7]—is criminalised in most regions of the world [8]. In addition to the social determinants described earlier, women who sell sex face a unique set of structural factors including police harassment and arrests, discrimination, marginalization, poverty, and gender inequality [8,9], as well as extreme occupational hazards such as violence, coercion, deception, alcohol and substance use, and human immunodeficiency virus (HIV)/sexually transmitted infection (STI) [10]. Together, these predispose female sex workers (FSWs) to increased psychological health vulnerabilities. Structural and occupational risks associated with sex work are highly dependent on sociocultural and economic contexts, which means that these hazards may differ for sex workers in LMICs and those in high-income countries. Evidence from high-income countries indicates a high prevalence of mental health morbidity among FSWs, especially post-traumatic stress disorder (PTSD), depression, anxiety, and psychological distress [11–14]. Three previous reviews have examined mental health in the context of STIs/HIV, alcohol use, and violence against sex workers [15–17]. However, no attempt has been made to date to synthesise the evidence or estimate the burden of mental health disorders for FSWs. This is vital to inform policy and programming at the global and country level. The aim of this systematic review is to estimate the prevalence of mental disorders among FSWs in LMICs, and to examine associations with factors that commonly affect their health and well-being (violence, alcohol and drug use, condom use, HIV/STI).

## Methods

### Search strategy and selection criteria

**Fig 1:**
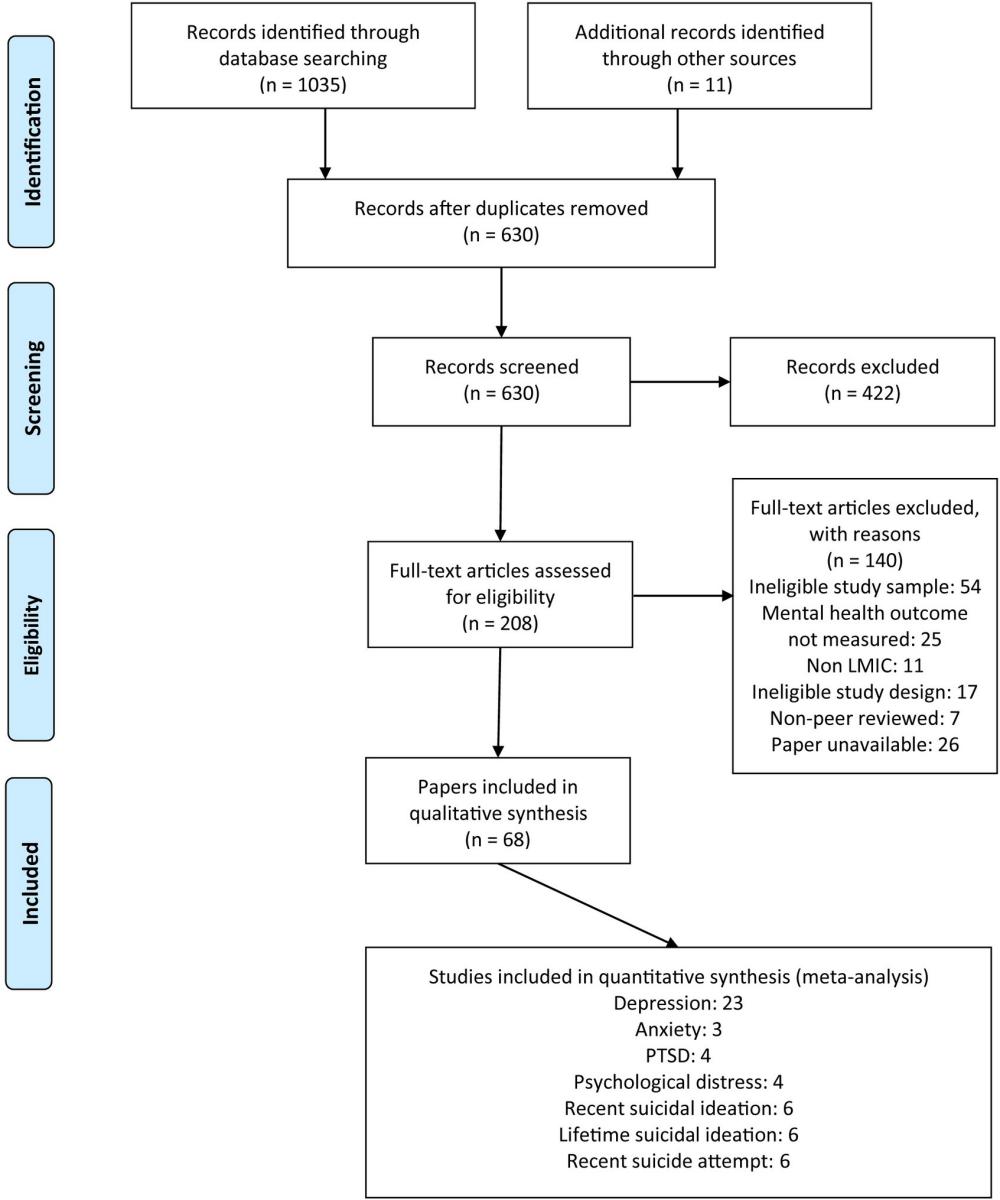
Flow chart of included quantitative studies. LMIC, low- and middle-income country; PTSD, post-traumatic stress disorder.

The review protocol has been registered with PROSPERO, number CRD42016049179 (https://www.crd.york.ac.uk/prospero/). Ethics approval was not required for this study. This study follows the Preferred Reporting Items for Systematic reviews and Meta-Analysis (PRISMA) guidelines (Fig 1; S1 Prisma Checklist). We searched electronic peer-reviewed literature databases (Ovid, PubMed, Web of Science) from first record until 26 April 2020. Search terms included the following: “mental health” OR “mental well-being” OR “psycholog* health” OR “psycholog* distress” OR “mental illness*” OR “mental disorder*” OR “mental health problem*” OR “psychiatr* morbidit*” OR “anxiety” OR “depress*” OR “suicid*” OR “trauma” OR “post-traumatic stress disorder” OR “PTSD”; “sex work*” OR “female sex work*” OR “prostitut*” OR “female prostitut*” OR “sex trad*” OR “transact* sex” OR “FSW*” OR “commercial sex” OR “sex-trade worker*”; “low and middle income countr*” OR “LAMIC*” OR “LMIC*” OR “developing countr*” OR “names of all countries which fit the LMIC criteria.” See S1 Text for full database list and search strategy.

Articles were included that measured the prevalence or incidence of mental health problems among FSWs, even if sex workers were not the main focus of the study. Only studies from countries defined as low or middle income, in accordance with the World Bank income groups 2019 [18], were included. Eligible studies had to be peer-reviewed, include females aged 16 or older who were actively engaged in sex work, and include the following study designs: cross-sectional survey, case–control study, cohort study, case series analysis, or experimental study with baseline measures for mental health. Studies were limited to English. We excluded studies that used qualitative methods only, were review papers, were conference abstracts, or were non–peer-reviewed publications. Studies exclusively focused on alcohol and substance use or victims of human trafficking sold into sex work were excluded from this review as reviews have recently been published in these areas [17,19–21]. Studies focused on women engaged in transactional sex only were ineligible for review, as this practice—and its implications on health—is distinct from sex work [22].

### Data extraction and quality assessment

All publications were screened by 2 independent reviewers (TB and BS). If either author classed a publication as relevant, the abstract was reviewed, with any disagreements discussed and a consensus reached. If eligibility could not be determined by screening of the title and abstract, the authors reviewed the full text. Three authors (BS, TB, and AM) assessed full texts using the eligibility criteria cited earlier, with any discrepancies resolved through discussion. Data were extracted by 3 authors (TB, BS, and AM) into a structured data extraction sheet.

Study quality was assessed by authors (TB, BS, and AM) using the Centre for Evidence-Based Management (CEBM) Critical Appraisal for Cross-Sectional Surveys Tool (S2 Text). One item on CEBM was removed (Item 12: “Can the results be applied to your organisation?”) as it was not applicable to this review. To assess quality of studies, authors rated each article on 11 items, and an overall score was calculated, with higher scores indicating stronger quality. Studies scoring ≥7 out of 11 points were considered strong quality, between 5 and 7 were rated moderate quality, and ≤4 were scored as weak quality. Individual scores are presented in Table 1, and detailed scoring of each study is presented in S3 Text.

**Table 1:** Studies and mental health outcomes.

Author and year	Country/income classification	Sample demographics	Sampling procedure	Outcome(s) of interest	Method of assessing outcome(s)	Events	Sample size	Prevalence (%)	Research quality
**AFRICA**									
Abelson (2019)	Cameroon, lower-middle income	FSWs Sex work location: Brothel, lodge, bar, other entertainment establishment: 100% Median age: 30.1 y (range: 23–36) Education: Primary/less: 30.9% Secondary/higher: 69.0% Martial status: Never married: 77.3% Currently married/in relationship: 10.7% Divorced/separated/ widowed: 12.0%	Respondent driven sampling	Depression	PHQ-9 with cut-off > 5	1,067	2,165	Any level depression: 49.8%	Strong (7)
Akinnawo (1995)	Nigeria, lower-middle income	FSWs Sex work location: Brothel, lodge, bar, other entertainment establishment: 100% Age: 30.5 y (range: not reported) Education: not reported Married status: Never married: 41.6% Currently married/in relationship: 44.0% Divorced/separated/ widowed: 14.4%	Purposive	Affection/mood disorder	Awaritefe Psychological Index	36	125	28.8%	Moderate (5)
				Neuroticism	Eysenck Personality Inventory	25		20.0%	
Barnhart (2019)	Tanzania, low income	Female bar workers Demographics not reported for FSWs	Random sampling	Depression	PHQ-9 measuring moderate to severe using cut-off > 10	3	23	13.0%	Moderate (6)
				PTSD	Primary care PTSD screening tool using cut-off >3	3		13.0%	
Berger (2018)	Swaziland, lower-middle income	FSWs Sex work location: Brothel, lodge, bar, other entertainment establishment: 27.0% Street/public place: 8.7% Home: 57.8% Mean age: 26 y (range: 16–49) Education: Primary/less: 32.4%, Secondary/higher: 67.9% Martial status: Never married: 89.1%, Currently married/in relationship: 4.1% Divorced/separated/widowed: 6.9%	Respondent driven sampling	Ever suicide ideation	“Have you ever felt like you wanted to end your life?”	188	325	58.6%	Moderate (5)
Bitty-Anderson (2019)**, Tchankoni (2020)**	Togo, low income	FSWs Sex work location: Brothel, lodge, bar, other entertainment establishment: 100% Median age: 25 y, IQR 21–32 y Education: Primary/less: 44.9% Secondary/higher: 55.1% Marital status: Never married: 86.1% currently married/in relationship: 13.9% Divorced/separated/widowed: 0%	Respondent driven sampling	Psychological distress	Kessler Psychological Distress Scale (K10) with cut-off scores: Mild: 20–24 Moderate: 25–29 Severe: >30	Mild: 223 Severe/moderate: 181	952	Mild: 23.4% Severe/moderate: 19%	Moderate (6)
Cange (2019)	Burkina Faso, low income	FSWs Sex work location: not reported Mean age: 26 y (range: 18–59) Education: Primary/less: 53.8% Secondary/higher: 0.7% Martial status: Never married: 53.6% Currently married/in relationship: 10.9% Divorced/separated/widowed: 35.5%	Respondent driven sampling	Ever depression	“Ever felt sad or depressed for more than 2 weeks at a time”	290	695	41.8%	Moderate (5)
				Ever suicide ideation	Ever wanting to end their life at least once	149		21.4%	
Coetzee (2018)	South Africa, upper-middle income	FSWs Sex work location: not reported Median age: 31 y (IQR: 25–37) Education: Primary/less: 75.6% Secondary/higher: 12.4% Relationship status: not reported	Respondent driven sampling	Depression	CES-D 20 using cut-off >21	349	508	68.7%	Strong (7)
				PTSD	PTSD-8 using cut-off >9	201		39.6%	
				Suicide ideation in past month and year	“In the past month has the thought of ending your life been in your mind?” “Within the past year have you felt suicidal because you are a sex worker?”	15		2.9%	
				Suicide attempt in past year	“In past year have you attempted suicide?”	5		1%	
Grosso (2019)	Togo and Burkina Faso, low income	FSWs and MSM Sex work location: not reported Median age: not reported Education: primary/higher: 57.0% Martial status: not reported	Respondent driven sampling	Ever suicide ideation	“Have you ever had suicidal thoughts?”	284	1,383	20.5%	Moderate (5)
Kim (2018)	Burkina Faso, low income	FSWs and MSM Sex work location: not reported Age (categories): <20: 10.2% 21–29: 37.8% >30: 52% Education: not reported Martial status: Never married: 44.5% Currently married/in relationship: 4.3% Divorced/separated/widowed: 34.0%	Respondent driven sampling	Ever suicide ideation	“Have you ever felt like you wanted to end your life?”	80	350	22.9%	Moderate (5)
Lion (2017)	South Africa, upper-middle income	FSWs who use methamphetamines Sex work location: not reported Age: 29 y (range not reported) Education: primary or lower not reported Martial status: not reported	Respondent driven sampling	Depression	PHQ-9 using cut-off >10	NA	53	NA	Moderate (6)
				PTSD	PTSD Breslau’s 7 items using cut-off >4	21		38.7%	
MacLean (2018)	Malawi, low income	FSWs Sex work location: Not reported Median age: 24 y (IQR: 22–28) Education: Primary/less: 17.0% Secondary/higher: 2.0% Martial status: Never married: 14.0% Currently married/in relationship: 4.0 Divorced/separated/widowed: 81.0%	Purposive	Depression	PHQ-9 using cut-off >10	16	200	8%	Strong (7)
				PTSD	PCL using cut-off >38 and >44	16		8%	
				Suicide ideation past 2 weeks	Used PHQ-9 item: “Have you had thoughts you would be better off dead” (passive ideation) and “of hurting yourself in some way” (active ideation)	6		3%	
Ortblad (2020)	Uganda, low income	FSWs Sex work location: Not reported Median age: 28 y (IQR: 24–32) Education: Primary/less: 55.7% Secondary/higher: 43.1% Marital status: Not reported	Random sampling	Depression	PHQ-9 using cut-off > 10	Uganda: 416	960	43.2%	Strong (10)
				Suicide ideation past 2 weeks	Used PHQ-9 item: “Have you had thoughts you would be better off dead or of hurting yourself in some way”	302		31.5%	
	Zambia, lower-middle income			Depression		Zambia: 441	965	45.7%	
				Suicide ideation past 2 weeks		540		56.7%	
Peitzmier (2014)* Sherwood (2015)*	The Gambia, low income	FSWs Sex work location: Not reported Mean age: 31 y (Range: 17–51) Education: Primary/less: 60.4% Secondary/higher: 39.6% Martial status: ever married: 23.0% Currently married/in relationship: 2.0% Divorced/separated/widowed: 69.8%	Purposive	Depression	Sad or depressed mood for 2 or more weeks at a time in the past 3 years	154	246	62.6%	Moderate (6)
Poliah (2017)	South Africa, upper-middle income	FSWs Sex work location: Not reported Mean age: not reported Education: Primary/less: 65.2% Secondary/higher: 34.8% Martial status: Never married: 94.8% Currently married/in relationship: 2.6% Divorced/separated/widowed: 1.9%	Purposive	Depression	PHQ-9 using cut-off score >5	121	150	80.9%	Moderate (5)
				Depression and anxiety	SRQ-20 using cut-off score >7	120		78.4%	
Rhead (2018)	Zimbabwe, lower-middle income	FSWs Sex work location: Not reported Age range: 19–58 y Education: Primary/less: 42.5% Secondary/higher: 57.5% Marital status: Never married: 4.6% Currently married/in relationship: 50.0% Divorced/separated /widowed: 44.8%	Random sampling of venues followed by respondent driven sampling	Psychological distress	Shona Symptom Questionnaire Cut-off not reported	43	174	24.7%	Moderate (6)
Roberts (2018)	Kenya, lower-middle income	HIV-negative FSWs Sex work location: Brothel, lodge, bar, other entertainment establishment: 88.0% Street/public place: 0% Home: 12.1% Median age: 33.5 y (IQR: 27.2–40.6) Education: 3.1 y (IQR: 1.2–9.8) Marital status: not reported	Purposive	Depression	PHQ-9 using cut-off score >10	30	283	10.6%	Moderate (6)
				PTSD	PCL-C using cut-off score >30	*63*		22.1%	
**EASTERN MEDITERRANEAN**									
Lari (2014)	Iran, upper-middle income	FSWs with history of drug use who are engaged with harm reduction centres Sex work location: not reported Mean age: 32.5 y (range: 16–51) Education status: only reported for secondary: 39.2% Marital status: only reported for divorced: >50.0%	Nonprobability sample	Psychological distress	Symptom Checklist-90 Cut-off not reported	NA	125	NA	Weak (4)
Ranjbar (2019)	Iran, upper-middle income	FSWs Sex work location: not reported Age: 30.9 y (range: 18–45) Education and relationship status: not reported	Purposive	Mental health disorders	GHQ-28 using cut-off score >23	30	48	62.5%	Weak (4)
					Structured Clinical Interview for DSM-IV mood disorders	16		53.5%	
					Structured Clinical Interview for DSM-IV anxiety disorders	11		36.7%	
**EUROPE**									
Lang (2011)	Armenia, lower-middle income	FSWs Sex work location: Street/public place: 100% Median age: 33.8 y (range: 20–52) Education and relationship status: not reported	Purposive	Depressive symptoms	CES-D-8 item using cut-off >7	53	117	45%	Moderate (5)
**SOUTH EAST ASIA**									
Ghose (2015)	India, lower-middle income	HIV-positive FSWs attending an HIV clinic Sex work location: Brothel, lodge, bar, other entertainment establishment: 92% Street/public Place: 8% Mean age: 23 y Education and relationship status: not reported	Purposive	Depression	Hospital Anxiety Depression Scheme Cut-off not reported	30	100	30%	Moderate (5)
				Anxiety		44		44%	
Hengartner (2015)	Bangladesh, lower-middle income	FSWs Sex work location: Brothel, lodge, bar, other entertainment establishment: 71.8% Home: 61.8% Mean age: 23.2 y (range 11–48) Education: Yes: 23.0%; No: 77.0% Marital status: Currently married/in relationship: 23.2%	Response driven sampling	Major depressive disorder	WHO Mental Health Composite International Diagnostic Interview Cut-off not reported	11	259	4.2%	Moderate (6)
				Generalised anxiety disorder		54		20.8%	
				PTSD		8		3.1%	
Iaisuklang (2017)	India, lower-middle income	FSWs enrolled in an HIV programme Sex work location: not reported Mean age: 29.5 y Educational status: Primary/less: 24.0% Secondary/higher: 45.0% Martial status: Never married: 9.0% Currently married/in relationship: 34.0% Divorced/separated/widowed: 57.0%	Purposive	Major depressive disorder	MINI International Psychiatric Interview cut-off not reported	9	100	9%	Weak (4)
				Generalised anxiety disorder		8		8%	
				PTSD		21		21%	
Pandiyan (2012)	India, lower-middle income	FSWs who use alcohol or drugs, attending psychiatric outpatient department Demographics not reported	Purposive	Depression	GHQ items not specified Cut-off not reported Clinical interview to confirm diagnosis	142	200	71%	Weak (3)
				Anxiety		84		42%	
Patel (2016)	India, lower-middle income	FSWs Sex work location: Brothel, lodge, bar, other entertainment establishment: 27.6%, street/public place: 4.5%, Home: 13.9%, Mobile phones: 54% Mean age: 31.0 y Education: Yes: 43.8%, No: 56.2% Marital status: Never married: 5.0% Currently married/in relationship: 66.5% Divorced/separated/widowed: 28.5%	Random sampling	Depression	PHQ-2 using cut-off >3	696	2,400	29%	Strong (9)
Patel (2015)	India, lower-middle income	FSWs Sex work location: Brothel, lodge, bar, other entertainment establishment: 7.7% Street/public place: 63.8%, Home: 28.5% Age, education, and relationship status: not reported	Random sampling	Depression	PHQ-2 using cut-off >3	778	1,986	39.2%	Strong (9)
Shahmanesh (2009)	India, lower-middle income	FSWs Sex work location: Brothel, lodge, bar, other entertainment establishment: 57.6% Street/public place: 22.8% Home: 28.1% Mean age: not reported Education: Yes: 32.7%, No: 67.3% Martial status: Never married: 28.4% Currently married/in relationship: 31.3% Divorced/separated/widowed: 40.3%	Respondent driven sampling	Depression and anxiety	Kessler-10 Cut-off not reported	NA	326	NA	Strong (7)
				Suicide ideation past 3 months	Suicide items not described	114		34.9%	
				Suicide attempts past 3 months		61		18.7%	
Suresh (2009)	India, lower-middle income	FSWs Sex work location: Street/public place: 100% Mean age: 34 y (range: 20–27) Educational: Secondary: 51% Marital status: not reported	Purposive	Depression	CES-D cut-off not reported	49	57	86%	Weak (4)
				PTSD	PCL-C using cut-off >45	31		56%	
				Ever suicide ideation	Ever having thoughts of suicide at the level of forming a plan	17		30%	
**WESTERN PACIFIC**									
Brody (2016)	Cambodia, low income	Female entertainment workers Sex work location: Brothel, lodge, bar, other entertainment establishment: 100.0% Mean age: 25.6 y Education: Formal education: 6.4 y (mean) Marital status: Never married: 44.1% Currently married/in relationship: 28.6% Divorced/separated/widowed: 27.2%	Two-stage cluster sampling method	Psychological distress	GHQ-12: mean score for whole sample used as cut-off	284	657	43.2%	Strong (8)
				Suicide ideation past 3 months	Suicide items not described	128		19.5%	
				Suicide attempts past 3 months		48		7.3%	
Carlson (2017)	Mongolia, lower-middle income	FSWs with harmful level of alcohol use Demographics not reported	Purposive	Depression	BSI depression subscales using cut-off >63	134	222	60.4%	Moderate (5)
Chen (2017)	China, upper-middle income	FSWs working in commercial sex venues Sex work location: Brothel, lodge, bar, other entertainment establishment: 100% Mean age: 25.2 y (range: 18–42) Education: Secondary/less: 7.4% Secondary/higher: 25.5% Marital status: Never married: 30.2% Currently married/in relationship: 63.2% Divorced/separated/widowed: 6.0%	Random sampling venues and purposive selection of FSWs	Depression	CES-D 20 using cut-off score >16	189	457	41.3%	Moderate (5)
Gu (2010a)	China, upper-middle income	FSWs who inject drugs Sex work location: Brothel, lodge, bar, other entertainment establishment: 100% Mean age: not reported Education: Secondary/below: 75.5% Secondary/higher: 24.5% Marital status not reported	Snowball sampling	Depression	Depression subscale of Chinese Depression Anxiety Stress Scale Cut-off not reported	NA	234	NA	Moderate (6)
				Hopelessness	Chinese Hopelessness Scale Cut-off not reported	NA		NA	
Gu (2010b)	China, upper-middle income	FSWs who inject drugs Sex work location: not reported Mean age: 28.1 y Education: Primary/less: 17.6% Secondary/higher: 82.4% Marital status: Never married: 53.7% Currently married/in relationship: 19.0% Divorced/separated/widowed: 26.4%	Convenience	Psychological Distress	"You hate yourself very much"	155	216	71.8%	Moderate (5)
					"You feel very depressed"	167		77.3%	
					"You are suffering from severe insomnia"	142		65.7%	
Gu (2014)	China, upper-middle income	FSWs who inject drugs Sex work location: Brothel, lodge, bar, other entertainment establishment: 100% Mean age: 33.9 y Education: Primary/less: 13.6% Secondary/higher: 86.4% Marital status: Never married: 36.0% Currently married/in relationship: 46.5% Divorced/separated/widowed: 17.5%	Snowball sampling	Depression	Depression subscale of Chinese Depression Anxiety Stress Scale using cut-off >21	78	200	39.0%	Moderate (6)
				Suicide ideation past 6 months	"Have you thought of committing suicide in the past 6 months?"	89		44.7%	
				Suicide attempt past 6 months	“Have you attempted to commit suicide in the past 6 months?”	54		26.8%	
Hong (2010)	China, upper-middle income	FSWs Sex work location: Brothel, lodge, bar, other entertainment establishment: 100% Median age: 22.5 y (range: 16–42) Education: <6 y: 33.6%, 7–9 y: 48.1% >9 y: 29.6% Marital status: Not married: 85.5%, Currently married/in relationship: 14.5%	Purposive	Depression	CESD-10 using cut-off score of >16	94	310	30.3%	Moderate (6)
				Suicide ideation past 6 months	*“*In the past 6 months, have you thought of committing suicide?”	55		17.8%	
				Suicide attempt past 6 months	“In the past 6 months, have you attempted to commit suicide?”	28		9.0%	
Hong (2007a)***, Fang (2007)***, Wang (2007)***	China, upper-middle income	FSWs Sex work location: Brothel, lodge, bar, other entertainment establishment: 100.0% Mean age: 23.5 y Education (y): 5.8 y Marital status: Never married: 60.0% Currently married/in relationship: 35.2% Divorced/separated/widowed: 4.9%	Random sampling venues and purposive selection of FWSs	Suicide ideation past 6 months	*“*In the past 6 months, have you thought of committing suicide?”	40	454	14.2%	Strong (7)
				Suicide attempt past 6 months	“In the past 6 months, have you attempted to commit suicide?”	23		8.4%	
Hong (2007b)	China, upper-middle income	FSWs Sex work location: Street/public place: 100% Mean age: 23.5 y Education (y): 5.84 y (mean) Martial status: Never married: 57.5% Currently married/in relationship: 35.2% Divorced/separated/widowed: 6.9%	Purposive	Depression	CES-D 10 using cut-off score ≥16	174	278	62.6%	Moderate (6)
Hong (2013)^**†**^, Su (2014)^**†**^, Zhang (2014a)^**†**^, Zhang (2014b)^**†**^, Zhang (2017)^**†**^	China, upper-middle income	FSWs Sex work location: Brothel, lodge, bar, other entertainment establishment: 91.2%, Mini hotels and streets: 8.7% Mean Age: 24.8 y Education status: Primary/less: 63.4% Secondary/higher: 36.6% Marital status: Never in relationship: 71.5% Ever in relationship: 28.5%	Random sampling venues and purposive selection of FSWs	Depression	CES-D 10 using cut-off score ≥16	502	1,022	49.1%	Strong (8)
				Severe suicide ideation or suicide attempt	Ever “seriously considered killing yourself” or ever “tried to kill yourself”	97		9.5%	
				Ever suicide ideation	Ever “seriously considered killing yourself	83		8.0%	
				Ever suicide attempt	Ever “tried to kill yourself”	49		4.8%	
Huang (2014)^**§**^, Zaller (2014)^**§**^, Yang (2018)^**§**^	China, upper-middle income	FSWs Sex work location: Brothel, lodge, bar, other entertainment establishment: 100.0% Median age: 23.5 y (IQR 20.9–26.4 y) Education: Secondary/less: 56.7% Secondary/higher: 43.5% Marital status: Currently married/in relationship: 44.5%, Single/divorced/widowed: 54.4%	Random sampling venues and purposive selection of FSWs	Depression	CES-D using cut-off >20	34	154	22.1%	Strong (7)
				Anxiety	Social anxiety scale using cut-off >60	6		3.9%	
				Suicide ideation past year	Suicide ideation item not described	15		9.7%	
				Suicide attempt past year	Suicide attempt item not described (authors noted having plan as indicating an attempt)	9		5.8%	
				Suicidal behaviour past year	Suicide behavior not described	8		5.2%	
Jackson (2013)	China, upper-middle income	FSWs Sex work location: Brothel, lodge, bar, other entertainment establishment and street/public place: 100% Mean age: 26.7 y Education: Primary/less: 13.9% Secondary/higher: 86.9% Marital status: Currently married/in relationship: 64.4%	Purposive	Depression	CES-D cut-off not reported	NA	395	NA	Weak (4)
Muth (2017)	Cambodia, lower-middle income	HIV positive FSWs Sex work location: Brothel, lodge, bar, other entertainment establishment: 100.0% Median age: 32 y (IQR: 28–35) Education: Primary/less: 85.0% Secondary/higher: 15.0% Martial status: not reported	Purposive	Psychological distress	Kessler-10 cut-off not reported	27	88	31%	Moderate (6)
Offringa (2017)	Mongolia, lower-middle income	FSWs Sex work location: Brothel, lodge, bar, other entertainment establishment and street/public place: 100% Mean age: 35.2 y Education: Primary/less: 7.8% Secondary/higher: 92.2% Martial status: Divorced/separated/widowed: 52.3%	Random sampling	Depression	BSI depression subscale Cut-off not reported	NA	204	NA	Strong (7)
Sagtani (2013)	Nepal, low income	FSWs Demographics not reported	Snowball sampling	Depression	CES-D 20 using cut-off >16	173	210	82.4%	Strong (8)
Shen (2016)	China, upper-middle income	FSWs working in commercial sex venues Sex work location: Brothel, lodge, bar, other entertainment establishment and street/public place: 100% Mean age: not reported Education: Primary/less: 23.4% Secondary/higher: 76.5% Marital status: Not married: 42.3% Currently married: 43.6%	Convenience sampling	Depression	GHQ-12 Chinese version using sample mean score as cut-off	342	653	52.4%	Moderate (6)
Shrestha (2017)	Nepal, low income	FSWs (and MSM/trans) Sex work location: Brothel, lodge, bar, other entertainment establishment and street/public place: 100% Mean age: not reported Education by literacy status: Yes: 67.8% No: 32.1% Marital status: Currently married/in relationship: 59.0%	Random sampling	Depression	CES-D using cut-off >22	112	610	18.3%	Strong (7)
				Ever suicide ideation	Suicide item not described	210		4.4%	
Urada (2013)	Philippines, lower-middle income	FSWs Sex work location: Brothel, lodge, bar, other entertainment establishment: 100.0% Median age: 22 y (IQR: 20–25) Education: 10 y (IQR 9–10) Martial status: Currently married/in relationship: 29.0% Living alone/separated/widowed: 71%	Purposive	Depression	CES-D 24 using cut-off >23	83	143	58%	Moderate (6)
Witte (2010)	Mongolia, lower-middle income	FSWs who had recent unprotected sex with client, enrolled in National AIDs Foundation Program Sex work location: not reported Mean age: 28 y (range 18–40) Education: Secondary/higher: 100% Marital status: Never married: 67.0%	Purposive	Depression	BSI-depression subscale Cut-off score not reported	19	48	38%	Weak (4)
Yang (2005)	China, upper-middle income	Female migrants engaged in sex work Sex work location: Brothel, lodge, bar, other entertainment establishments: 100.0% Mean age: 23.9 y (mean) Education: Secondary/less: 92.3% Marital status: Never married: 76.9%	Random sampling of venues and convenience sample of FSWs	Depression	CES-D 20 using cut-off >16	20	40	50%	Moderate (6)
**AMERICAS**									
Devóglio (2017)	Brazil, upper-middle income	FSWs Sex work location: not reported Mean age: 26.8 y Education: Secondary/less: 30.1% Higher than secondary: 92.6% Relationship status: Never married: 94.0% Currently married/in relationship: 6.0% Divorced/separated/widowed: 4.8%	Purposive	Depression	Hospital Anxiety and Depression scale Cut-off not reported	11	83	13.2%	Moderate (5)
				Anxiety		33		39.7%	
González-Forteza (2014)	Mexico, upper-middle income	FSWs Sex work location: Brothel, lodge, bar, other entertainment establishment: 48% Street/public place: 16% Median age: 28.8 y Education: Primary/less: 31.4% Secondary/higher: 60.8% Marital status: Never married: 49.0% Currently married/in relationship: 28.0% Divorced/separated/widowed: 22.3%	Purposive	Depression	MINI International Psychiatric Interview, including suicide risk	41	103	39.8%	Weak (3)
				Ever suicide risk	MINI International Psychiatric Interview, including suicide risk: “Do you ever feel like life is not worth living?” “Have you ever thought about killing yourself. If so, how would you do it?”	40		38.8%	
Jain (2019)	Mexico, upper-middle income	FSWs Sex work location: not reported Median age: 38 y (IQR: 20–46) Education: Secondary: 44.1% Marital status: not reported	Purposive	Depression	Beck Depression Inventory using cut-off score >20	106	295	35.9%	Moderate (5)
Logie (2018)	Jamaica, upper-middle income	FSWs who are lesbian and bisexual women Sex work location: not reported Mean age: 27.2 y (range: 19–43) Education: not reported Marital status: Never married: 8.9% Currently married/in relationship: 71.1%	Purposive	Depression	PHQ-2 using cut-off >3	42	45	93.33%	Moderate (6)
Rael (2017a)^**‡**^, Rael (2017b)^**‡**^	Dominican Republic, upper-middle income	HIV-negative FSWs with dependent children Sex work location: Brothel, lodge, bar, other entertainment establishment: 70.1% Mean age: 27.5 y Education: 8.4 y (mean) Marital status: Currently married/in relationship: 32.4%	Purposive sample	Depression	CES-D 10 using cut-off >10	245	349	70.20%	Moderate (6)
Semple (2019)	Mexico, upper-middle income	FSWs in street-based work and establishment-based indoor sex work Sex work location: Brothel, lodge, bar, other entertainment establishment: 39.0% Street/public place: 61.0% Mean age: 33.6 y (range: 18–56) Education: Primary/less: 41.8%, secondary/higher: 59.1% Marital status: Never married: 56.0% Currently married/in relationship: 28.8% Divorced/separated/widowed: 14.7%	Time-location sampling	Depression	Beck Depression Inventory using cut-off score >20	155	426	36.4%	Moderate (6)
Ulibarri (2009)^Ꝁ^, Ulibarri (2014)^Ꝁ^	Mexico, upper-middle income	HIV-negative FSWs Sex work location: Brothel, lodge, bar, other entertainment establishment: 41.2% Street/public place: 54.8%, Other: 3.2% Mean age: 33.4 y (range 18–64) Education: 6.13 y (mean) Marital status: Never married: 46.0% Currently married/in relationship: 33.0% Divorced/separated/widowed: 26.0%	Purposive	Psychological distress (depression and somatization)	Brief Symptom Inventory Subscales: depression and somatization (cut-off not reported)	NA	916	NA	Moderate (5)
Ulibarri (2013)	Mexico, upper-middle income	FSWs who inject drugs Sex work location: not reported Mean age: 33.7 y Educational status: 7.1 y (mean) Martial status: Never married: 49.0% Currently married/in relationship: 38.0% Divorced/separated/widowed: 13.3%	Purposive	Depression	CES-D 10 using cut-off >10	538	624	86.2%	Moderate (6)
Ulibarri (2015)	Mexico, upper-middle income	FSWs who use drugs and have a regular partner Sex work location: not reported Mean age: 37.3 y (mean) Education: 6.7 y (mean) Marital status: Currently married/in relationship: 98.0%	Snowball sampling	Depression	CES-D 10 cut-off not reported	NA	214	NA	Moderate (5)

*Papers report findings on same study but explore different associations with outcome of interest.

**Papers report findings on same study but explore different associations with outcome of interest.

***Papers report findings on same study but explore different associations with outcome of interest.

^†^Papers report findings on same study but explore different associations with outcome of interest.

^§^Papers report findings on same study but explore different associations with outcome of interest.

^‡^Papers report findings on same study but explore different associations with outcome of interest.

^Ꝁ^Papers report findings on same study but explore different associations with outcome of interest.

**Abbreviations:** BSI, Brief Symptom Inventory; CES-D, Centre for Epidemiological Studies Depression Scale; DSM-IV, Diagnostic and Statistical Manual of Mental Disorders, 4th edition; FSW, female sex worker; GHQ, General Health Questionnaire; HIV, human immunodeficiency virus; IQR, interquartile range; MINI, Mini-International Neuropsychiatric Interview; MSM, men who have sex with men; NA, not applicable; PCL, PTSD CheckList; PCL-C, PTSD CheckList – Civilian Version; PHQ, Patient Health Questionnaire; PTSD, post-traumatic stress disorder; SRQ, WHO Self-Reporting Questionnaire.

### Data analysis

A narrative synthesis was conducted across all studies meeting inclusion criteria. Prevalence estimates were calculated from percentages or raw proportions, and we contacted authors of studies in which raw data were missing. If multiple publications reported results from a single study, we included all studies in Table 1 but only the original study in the narrative synthesis and prevalence analyses. Meta-analyses were conducted on studies that scored moderate to strong in the quality assessment and that used validated measures to assess mental health outcomes; we excluded studies from the meta-analyses that sampled participants based on characteristics that are known to be an independent risk factor for mental health problems (such as injecting drug use or HIV status) and could therefore bias the pooled mental health estimates. Analyses were completed using Comprehensive Meta-Analysis (CMA) software version 3 (Biostat, Englewood, NJ). Pooled estimates were calculated using a random effects model. Variation between studies was determined by heterogeneity tests with the Higgins’ *I*^2^ statistic. Relative weights were calculated using the formula 1/V + T^2^ where V is the error variance and T^2^ (Tau-squared) is the between-study variance. Subgroup analyses were completed to examine associations between mental health outcomes (e.g., depression) and the following covariates: violence/police arrest, alcohol/drug use, condom use, and HIV/STI. Due to variations between studies in the factors adjusted for in multivariate analyses, unadjusted odds ratios (ORs) were extracted or calculated from raw data. Pooled effect estimates were calculated using a random-effects model.

## Results

### Study characteristics

The initial electronic search yielded 1,035 results, with 11 more studies identified through reference list screening and online searches. After duplicate records were removed, the titles and abstracts of 630 publications were screened for eligibility. Of those, 208 were identified as potentially relevant publications and reviewed for inclusion. Sixty-eight papers reporting on 56 unique studies with 24,940 participants meeting the inclusion criteria (Fig 1). Eight of these studies did not provide prevalence data on mental health [23–31]; authors of these studies were contacted twice for further information, and 2 authors responded, providing prevalence data [23,27]. In total, 86 prevalence estimates from 48 studies were available (depression *n* = 37; anxiety n = 7; PTSD *n* = 8; suicide attempt *n* = 8; suicide ideation *n* = 17; psychological distress *n* = 7; mood disorders *n* = 2) (Table 1).

Studies were based in 26 LMICs: 13 countries in sub-Saharan Africa, 1 in the Middle East and north Africa region, 1 in Eastern Europe, 2 in South East Asia, 5 in the Western Pacific region, and 4 in Latin America and the Caribbean. Eleven studies reported findings from countries in the low-income group, 20 studies from the low-middle income group, and 26 studies from the upper-middle income group, as per the World Bank income classification. Twenty-nine studies used a purposive sample, 14 used respondent driven sampling techniques, 7 used a random sample, and 6 utilized random sampling techniques to select venues and purposive methodology to recruit FSWs within these venues (Table 1). Most studies recruited FSWs from a variety of venues, such as streets, bars, brothels, and entertainment establishments, with 3 studies selecting women from clinics or hospital settings [26,32,33]. All studies were cross-sectional, with 3 studies including qualitative data alongside survey results [34–36]. Of the 56 studies, 14 scored as strong quality, 34 scored as moderate, and 8 scored as weak (S3 Text). Sixteen studies (14 moderate; 2 weak) selected participants based on harmful alcohol or drug use (*n* = 9) [24,26,31,33,34,37–40], or positive [32,41] or negative [29,42–45] HIV status (*n* = 5), and were excluded from the meta-analyses (regardless of CEBM score) to avoid biasing the pooled estimates. Analyses used a variety of validated scales and cut-off points to assess mental disorders (Table 1). None reported a mental health intervention.

The mean age of FSWs in the 42 studies that reported this was 28.9 years (age range: 11–64 years). Thirty-two studies reported sex work locations for their sample; among these studies, 66.3% of FSWs worked in brothels, lodges, bars, or other entertainment establishments; 51.7% worked in streets or public places; 24.7% worked at home; and 36.7% worked in other settings, e.g., via mobile phones (these categories were not mutually exclusive). Thirty-one studies reported education levels of their sample, and among these, nearly one-half of FSWs (45.7%) had an education level of primary school or less. Among the 40 studies that reported marital status for their sample, 48% of FSWs were never married; 32.9% were currently married or in a relationship; and 24.5% were divorced, separated, or widowed.

### Mental disorders and suicidal behaviour

**Fig 2:**
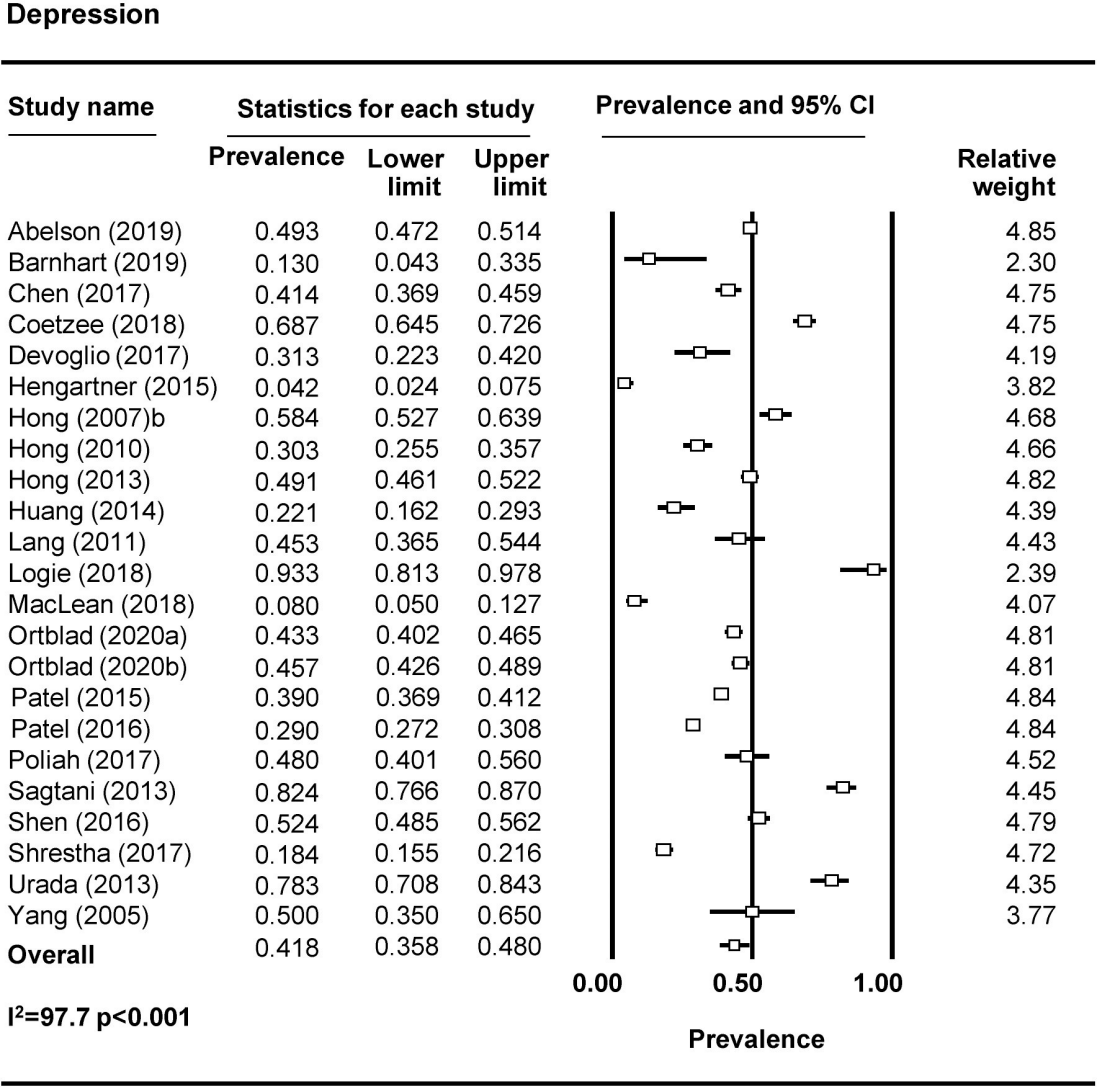
Depression pooled prevalence estimates.

**Fig 3:**
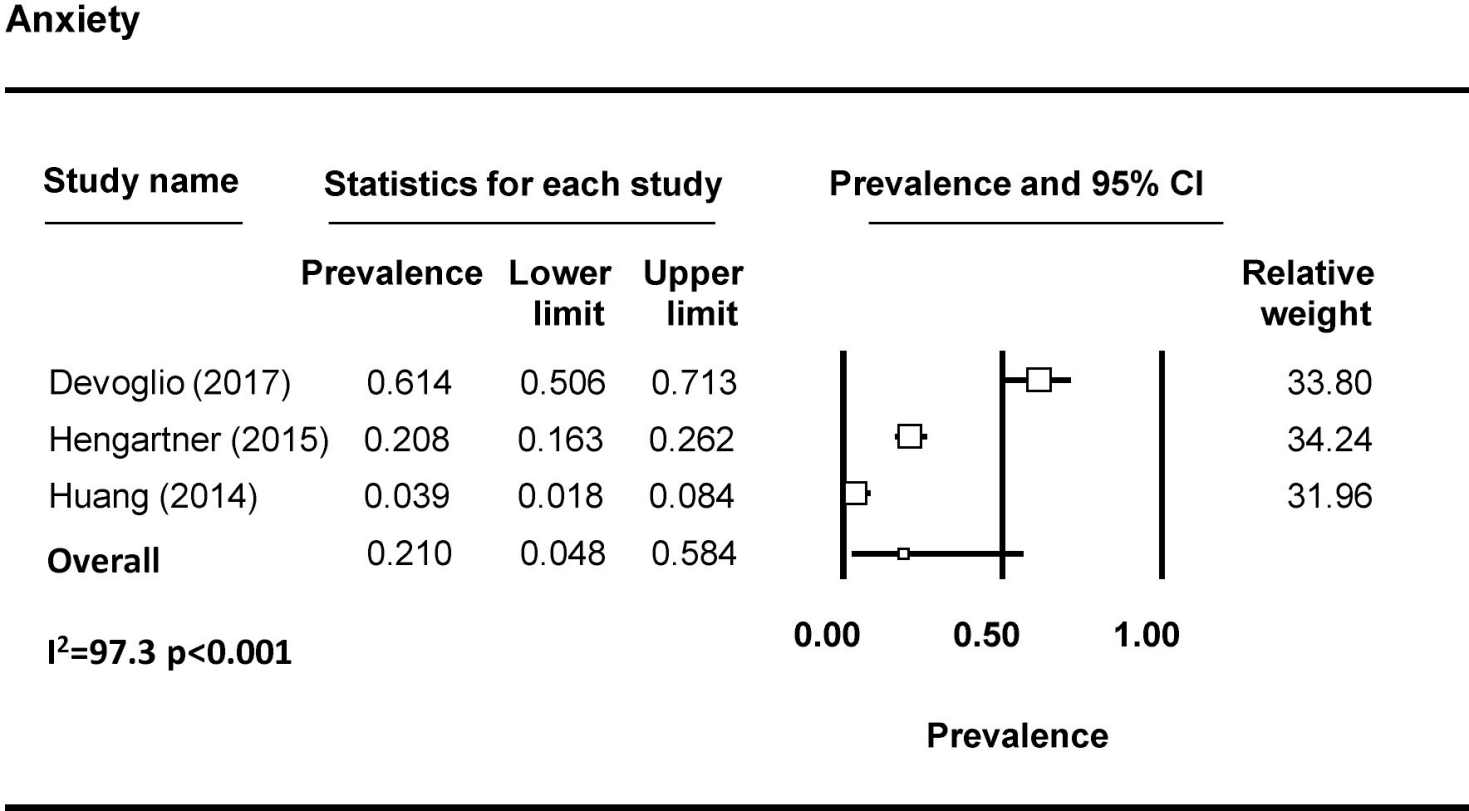
Anxiety pooled prevalence estimates.

**Fig 4:**
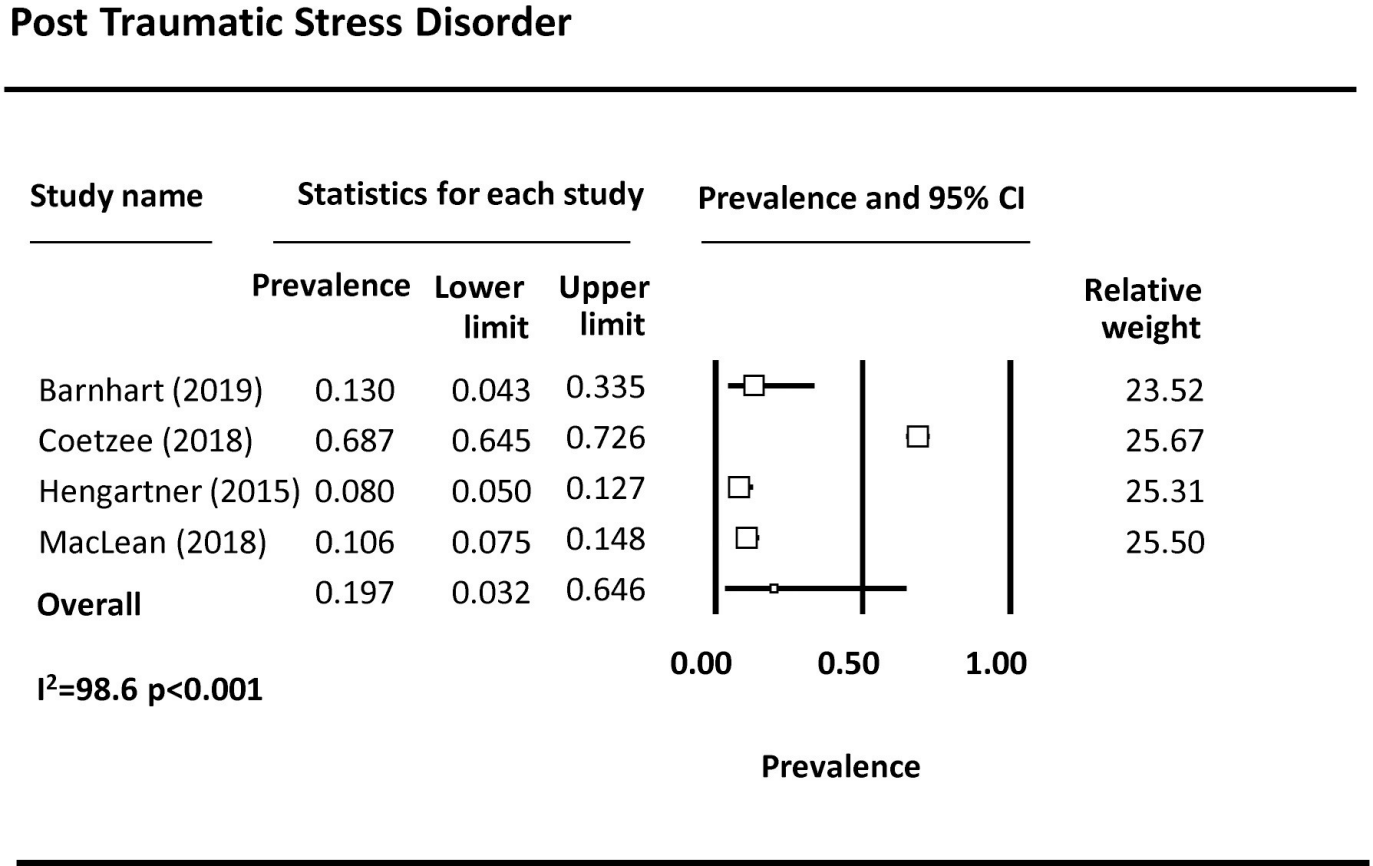
PTSD pooled prevalence estimates. PTSD, post-traumatic stress disorder.

**Fig 5:**
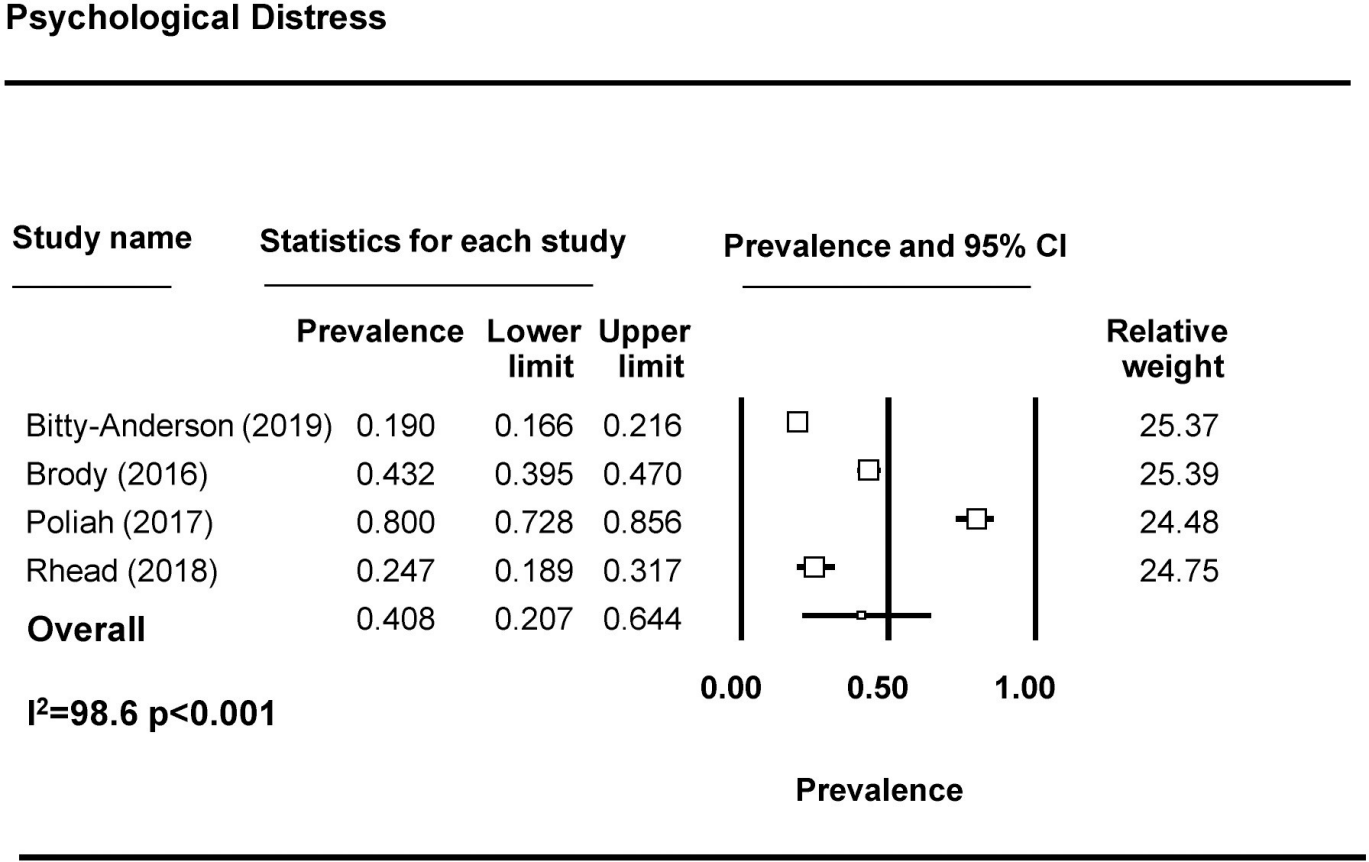
Psychological distress pooled prevalence estimates.

Forty-four studies examined depression among FSWs, with 37 reporting prevalence estimates (Table 1) [9,10,23,27,32–37,39,43–69]. A meta-analysis was conducted with 23 studies (Fig 2). The pooled prevalence of depression among FSWs from LMICs is 41.8% (95% CI 35.8%–48.0%). Seven studies reported on the prevalence of anxiety among FSWs [9,32,33,50,58,64,70], with 3 included in the meta-analysis (Fig 3). The pooled prevalence of anxiety among FSWs from LMICs is 21.0% (95% CI 4.8%–58.4%). PTSD symptomology was reported in 8 studies [9,10,23,40,43,46,47,50] with 4 studies included in the meta-analysis (Fig 4). The pooled prevalence of PTSD symptoms among FSWs from LMICs is 19.7% (95% CI 3.2%–64.6%). Ten studies measured psychological distress among FSWs, with 7 studies providing prevalence estimates [38,41,49,70–73] and 4 studies included in the meta-analysis (Fig 5). The pooled prevalence of psychological distress experienced by FSWs from LMICs was 40.8% (95% CI 20.7%–64.4%). Two studies examined mood disorders [70,74]. Only one study [74] was eligible for inclusion in a meta analysis and thus a pooled prevalence estimate is not available. This study reported a prevalence of affection/mood disorder of 28.8% (95% CI 21.5%–37.3%).

**Fig 6:**
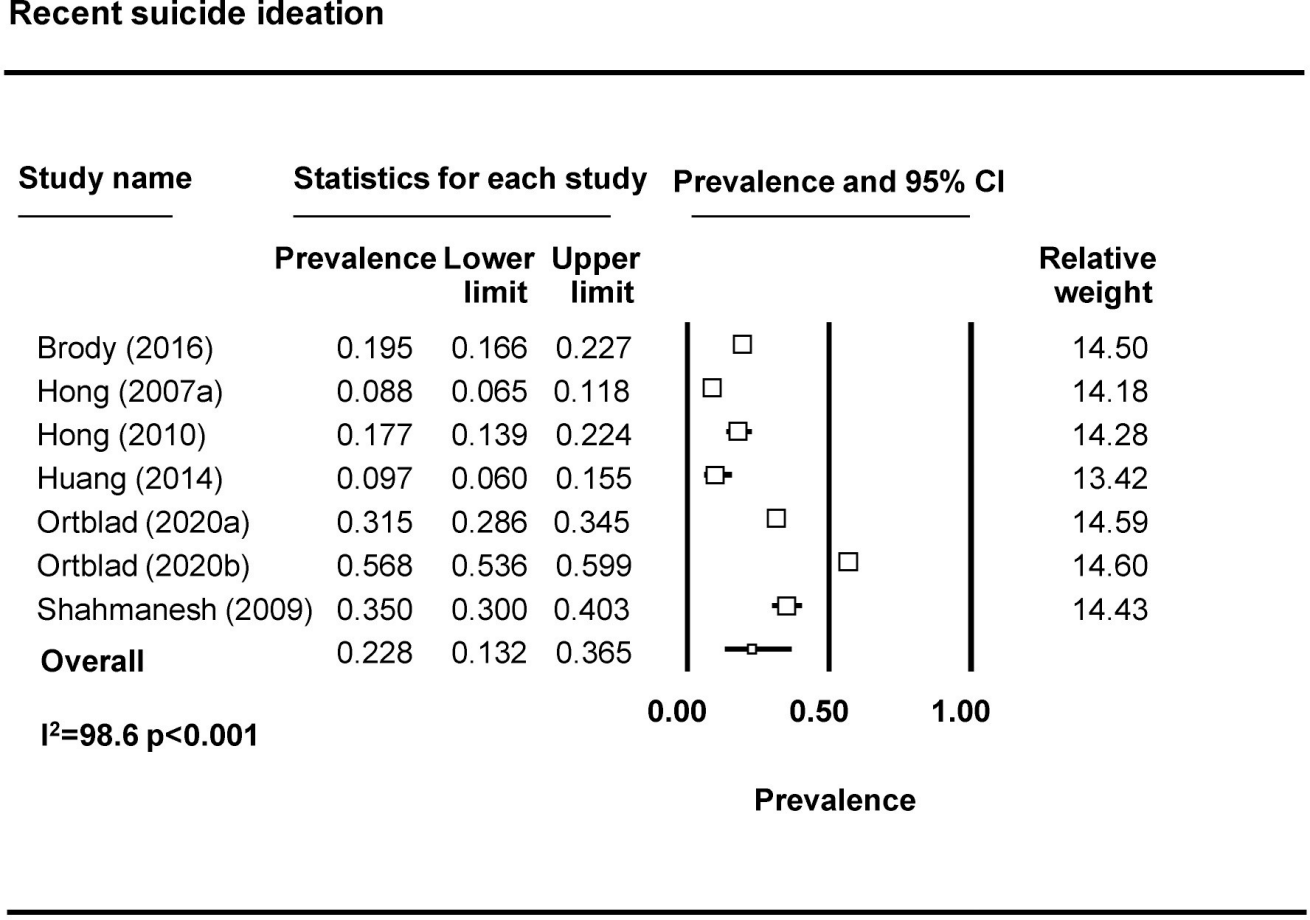
Recent suicide ideation pooled prevalence estimates.

**Fig 7:**
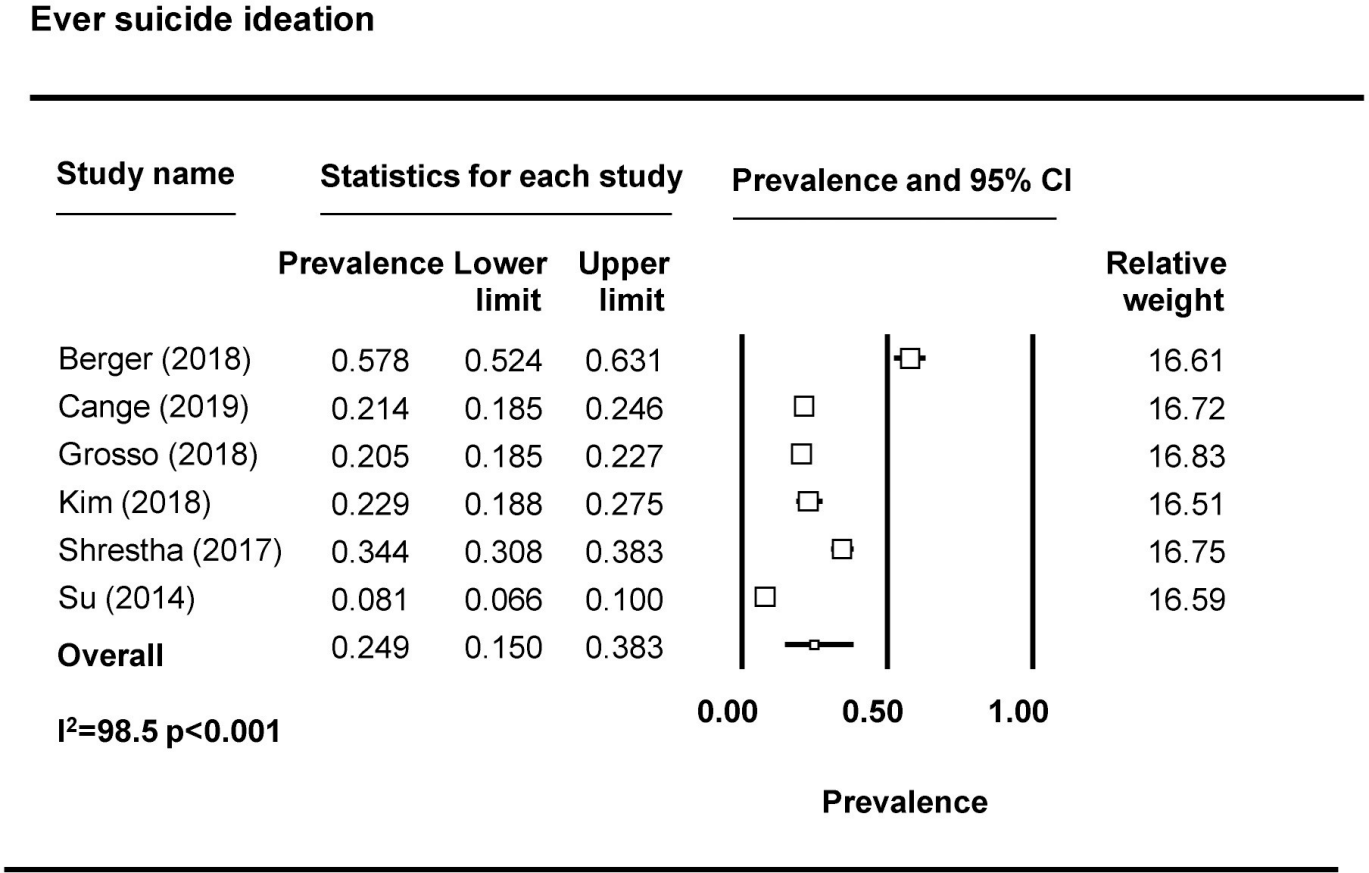
Ever suicide ideation pooled prevalence estimates.

**Fig 8:**
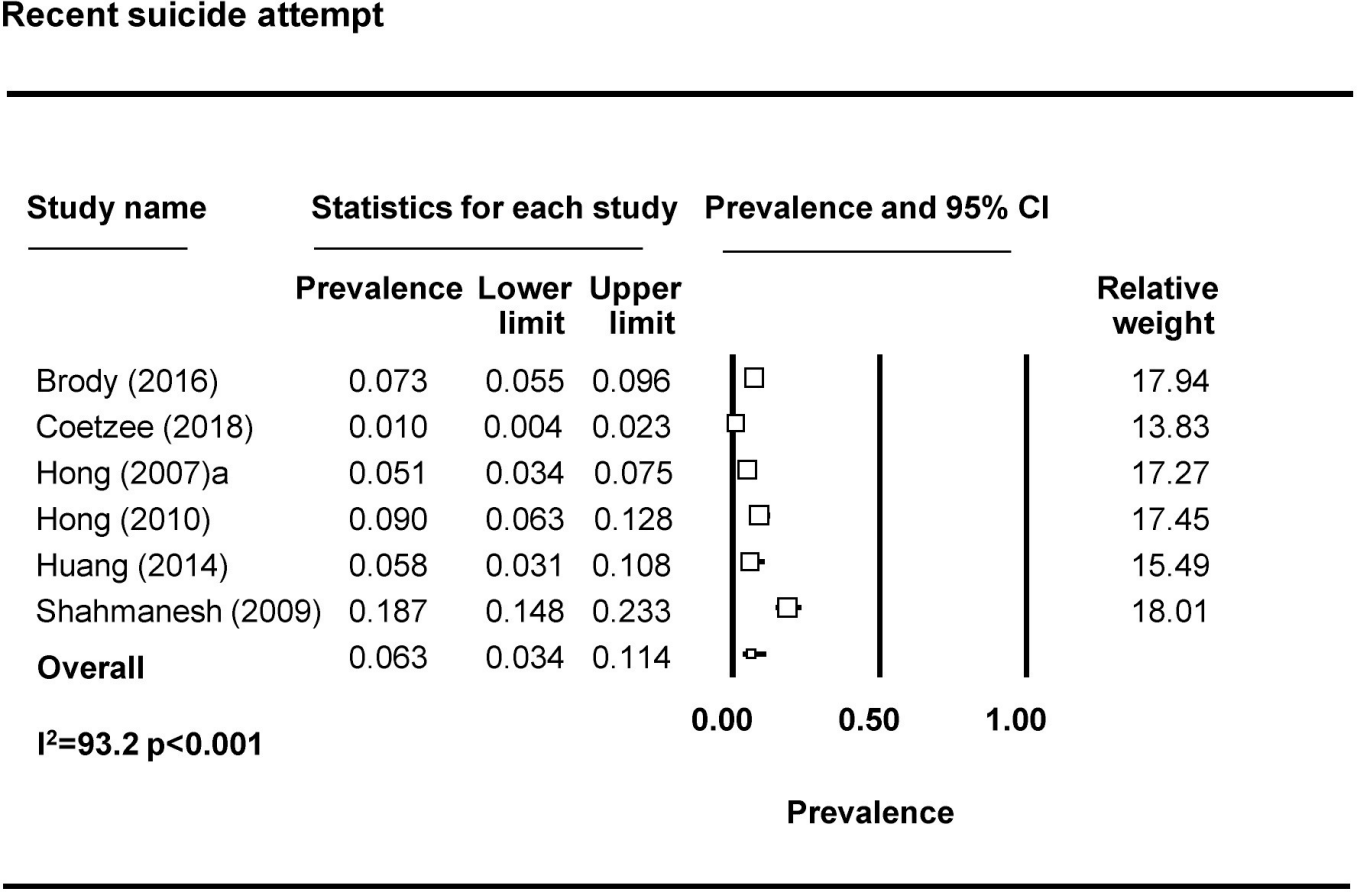
Recent suicide attempt pooled prevalence estimates.

Seventeen studies reported on suicidal ideation [10,36,39,46,47,55,57,58,61,69,71,75–80]. Most assessed suicidal ideation by asking about suicidal thoughts, for example, “have you thought about killing yourself?” and “have you ever felt like you wanted to end your life?” For the meta-analysis, we divided studies based on timeframe into ‘recent’ or ‘ever’ suicidal ideation and removed 3 studies due to limitations in how questions were operationalized [46,47], including one study that combined suicidal thoughts with attempting suicide [65]. The pooled prevalence of recent (past 3 months, 6 months, or year) suicide ideation is 22.8% (95% CI 13.2%–36.5%) (*n* = 6 studies from 7 countries) (Fig 6). The pooled prevalence of lifetime suicidal ideation is 24.9% (95% CI 15.0%–38.3%) (*n* = 6 studies) (Fig 7). Eight studies reported on suicide attempts among FSWs [39,46,55,57,58,71,79,80]. The majority assessed suicide attempts through one binary question (yes/no) asking whether the participant had attempted suicide. Prevalence of recent suicide attempt (past 3 months, 6 months, or year) was reported by 6 studies included in the meta-analysis (Fig 8). The pooled prevalence of recent suicide attempts among FSWs from LMICs is 6.3% (95% CI 3.4–11.4%). Only one study reporting on ever suicide attempt was eligible for inclusion in a meta analyses and thus a pooled prevalence estimate is not available. This study reported a prevalence of lifetime suicide attempt of 4.8% (95% CI 3.6%–6.3%) [81].

### Associations between mental health and other factors

We conducted subgroup analyses to examine associations between mental health (e.g., depression) and factors commonly experienced by FSWs (violence/police arrest, alcohol/drug use, condom use, and HIV/STI) (Table 2). Findings of the meta-analyses are summarised in Table 3 and displayed in forest plots in S1–S4 Figs.

**Table 2:** Studies on mental health and outcomes of interest.

Author and study	Country	Sample	Mental health measure	Outcome(s) of interest	Sample size	Odds in the exposed^1^	Odds in the unexposed^2^	Crude OR (95% CI)	*P* value
**VIOLENCE**									
Berger (2018)	Swaziland	FSWs	Suicide ideation ever	Physical violence (as a result of selling sex) or sexual violence ever	325	124/65	67/68	1.9 (1.2–3.0)	0.006
Cange (2019)	Burkina Faso	FSWs	Suicide ideation ever	Physical violence ever	696	106/43	318/220	1.7 (1.2–2.5)	0.008
				Sexual violence ever		91/58	193/353	2.9 (2.0–4.2)	<0.001
Carlson (2017)	Mongolia	FSWs with harmful level of alcohol use	Depression (BSI)	Physical violence ever by client	222	114/20	67/21	1.8 (0.9–3.5)	0.09
				Sexual violence ever by client		79/55	24/64	3.8 (2.1–6.9)	<0.001
Coetzee (2018)	South Africa	FSWs	Depression (CES-D)	Physical or sexual violence past year	508	295/41	142/28	1.4 (0.8–2.4)	0.2
			PTSD (PTSD-8)			244/31	195/38	1.5 (0.9–2.6)	0.13
Gu (2014)	China	FSWs who inject drugs	Depression (Chinese Depression Anxiety Stress Scale)	Verbal, physical violence or threats past 6 months by clients or gatekeepers	200	25/53	25/97	1.83 (1.0–3.5)	0.06
			Suicide ideation past 6 months			33/56	16/94	3.4 (1.6–7.0)	0.001
			Suicide attempt past 6 months			22/30	26/116	3.2 (1.3–7.6)	0.01
Hong (2007b)	China	FSWs	Depression (CES-D)	Sexual violence past 6 months	278	17/157	3/101	1.5 (0.4–5.9)	0.6
			Suicide ideation past 6 months			18/47	52/337	2.5 (1.3–4.6)	0.004
			Suicide attempt past 6 months			14/24	60/356	3.5 (1.7–7.1)	0.001
Hong (2013)* Zhang (2017)*	China	FSWs	Depression (CES-D)	Sexual, physical, or emotional violence ever by client	1,022	252/170	244/271	1.6 (1.3–2.1)	<0.001
				Sexual, physical, or emotional violence ever by intimate partner		241/189	124/189	1.9 (1.4–2.6)	<0.001
			Suicide ideation or attempt ever	Sexual, physical, or emotional violence ever by client		55/367	37/478	1.9 (1.2–3.0)	0.006
				Sexual, physical, or emotional violence ever by intimate partner		52/378	15/298	2.7 (1.5–5.0)	0.001
			Depression (CES-D)	Sexual, physical, or emotional violence ever by intimate partner or non-partner		358/163	279/222	1.7 (1.4–2.3)	<0.001
			Suicide ideation or attempt ever	Sexual, physical, or emotional violence ever by intimate partner or non-partner		79/18	558/367	2.9 (1.7–4.9)	<0.001
Jain (2020)	Mexico	FSWs	Depression (PHQ-9)	Physical violence ever by client	295	50/56	57/132	2.1 (1.3–3.4)	0.002
Patel (2015)	India	FSWs	Depression (PHQ-2)	Physical violence past 6 months	1,986	158/620	189/1020	1.4 (1.1–1.7)	0.008
				Police arrest ever		165/613	133/1075	2.2 (1.7–2.8)	<0.001
Patel (2016)	India	FSWs	Depression (PHQ-2)	Physical or sexual violence past year	2,400	285/418	291/1407	3.3 (2.7–4.0)	<0.001
Poliah (2017)	South Africa	FSWs	Depression (PHQ-9)	Violence ever during sex work	150	93/27	17/11	2.2 (0.9–5.3)	0.08
				Police harassment ever		85/38	19/8	0.9 (0.4–2.3)	0.9
Roberts (2018)	Kenya	HIV-negative FSWs	Depression (PHQ-9)	Sexual, physical, moderate emotional violence ever by intimate partner or non-partner	283	26/4	197/56	1.8 (0.6–5.5)	0.3
			PTSD (PCL-C)			41/5	182/55	2.5 (0.9–6.6)	0.07
Sagtani (2013)	Nepal	FSWs	Depression (CES-D)	Physical, sexual, or emotional violence past 6 months	210	90/62	10/48	7.0 (3.2–15.1)	<0.001
Shahmanesh (2009)	India	FSWs	Suicide attempt past 3 months	Sexual violence ever	326	18/55	18/234	4.3 (2.1–8.7)	<0.001
				Sexual, physical, or verbal violence past 12 months by intimate partner		38/55	29/44	2.3 (1.2–4.3)	0.01
				Sexual, physical, or verbal violence past 12 months by others		29/44	40/212	3.5 (2.0–6.2)	0.001
				Police raid past 12 months		18/55	32/220	2.3 (1.2–4.3)	0.01
Sherwood (2015)	Gambia	FSWs	Sad or depressed mood for more than 2 weeks at a time in past 3 years	Sexual violence ever by client	251	51/100	19/71	1.9 (1.0–3.5)	0.05
Ulibarri (2013)	Mexico	FSWs who inject drugs	Depression (CES-D)	Physical violence ever	624	269/269	35/49	1.4 (0.9–2.2)	0.1
				Physical violence ever by client		108/430	15/69	1.2 (0.6–2.1)	0.6
				Sexual violence ever		283/255	30/54	2.0 (1.2–3.2)	0.006
Ulibarri (2014)	Mexico	HIV-negative FSWs	Psychological distress (BSI)	Physical, sexual, or emotional violence past 6 months by clients	924	NA	NA	2.0 (1.6–2.4)	<0.001
**ALCOHOL AND DRUG USE**									
Bitty-Anderson (2019)*, Tchankoni (2020)*	Togo	FSWs	Psychological distress (Kessler)	Harmful/hazardous alcohol (AUDIT)	952	82/99	350/421	1.0 (0.7–1.4)	1.0
Coetzee (2018)	South Africa	FSWs	Depression (CES-D)	Severe binge drinking (AUDIT)	508	196/174	81/55	0.8 (0.5–1.1)	0.2
			PTSD (PTSD-8)			99/96	179/134	0.8 (0.5–1.1)	0.2
Hong (2007a)	China	FSWs	Depression (CES-D)	Alcohol intoxication past 6 months	454	62/112	26/78	1.7 (1.0–2.9)	0.05
			Suicide ideation past 6 months			31/34	118/221	1.7 (1.0–2.9)	0.06
			Suicide attempt past 6 months			21/17	128/288	2.8 (1.4–5.4)	0.003
Jain (2020)	Mexico	HIV-negative FSWs	Depression (PHQ-9)	Hazardous alcohol past year (AUDIT)	295	57/49	79/110	1.6 (1.0–2.6)	0.05
				Polydrug use past month		45/61	40/149	2.8 (1.6–4.6)	<0.001
Patel (2015)	India	FSWs	Depression (PHQ-2)	Alcohol use past 30 days	1,986	493/285	455/751	2.9 (2.4–3.4)	<0.001
Zhang (2014a)	China	FSWs	Depression (CES-D)	Illicit drug use ever	1,022	118/403	67/434	1.9 (1.4–2.6)	<0.001
Zaller (2014)***, Yang (2018)***	China	FSWs	Depression (CES-D)	Alcohol dependent (AUDIT ≥16)	358	19/32	76/231	1.8 (1.0–3.4)	0.06
			Suicide ideation past 12 months	Alcohol dependent (AUDIT ≥16)		11/84	22/241	1.4 (0.7–3.1)	0.4
			Depression (CES-D)	Illicit drug use ever		15/96	12/235	3.1 (1.4–6.8)	0.005
**CONDOM USE**									
Abelson (2019)	Cameroon	FSWs	Depression (PHQ-9)	Inconsistent condom use with clients ever	2,136	108/274	391/1363	1.4 (1.1–1.8)	0.013
Brody (2016)	Cambodia	FSWs	Psychological distress (GHQ-12)	Inconsistent condom use with clients past 3 months	657	235/49	322/51	0.8 (0.5–1.2)	0.2
				Inconsistent condom use with partner past 3 months		255/29	340/33	0.9 (0.5–1.4)	0.6
Gu (2010a)	China	FSWs who inject drugs	Depression (Chinese Depression Anxiety Stress Scale)	Inconsistent condom use with clients past 6 months	234	NA	NA	1.2 (1.1–1.3)	<0.001
Hong (2007b)	China	FSWs	Depression (CES-D)	Inconsistent condom use with clients	278	140/34	62/42	2.8 (1.6–4.8)	<0.001
Patel (2015)	India	FSWs	Depression (PHQ-2)	Inconsistent condom use occasional clients	1,986	274/504	277/928	1.8 (1.5–2.2)	<0.001
				Inconsistent condom use with regular clients		356/41	342/845	2.1 (1.8–2.6)	<0.001
Shahmanesh (2009)	India	FSWs	Suicide attempt past 3 months	Inconsistent condom use with clients	326	26/47	63/189	4.3 (2.1–8.7)	<0.001
Shen (2016)	China	FSWs	Depression (GHQ)	No condom last sex client	653	69/270	64/245	1.0 (0.7–1.4)	1.0
				No condom last sex partner		70/138	120/105	0.4 (0.3–0.7)	<0.001
Urada (2013)	Philippines	FSWs	Depression (CES-D)	Inconsistent condom use with clients	143	47/60	13/23	1.4 (0.6–3.0)	0.4
Zaller (2014)***, Yang (2018)***	China	FSWs	Depression (CES-D)	Inconsistent condom use with clients past 6 months	358	16/95	16/231	1.6 (1.2–2.1)	0.018
				Inconsistent condom use with partner past 6 months		67/44	138/109	1.2 (0.9–1.9)	0.4
**HIV/STIs**									
Bitty-Anderson (2019)*, Tchankoni (2020)*	Togo	FSWs	Psychological distress (Kessler)	HIV positive	952	80/101	45/726	12.8 (8.4–19.5)	<0.001
Cange (2019)	Burkino Faso	FSWs	Suicide ideation ever	HIV positive	696	22/104	56/395	1.5 (0.9–2.6)	0.14
Jain (2020)	Mexico	HIV-negative FSWs	Depression (PHQ-9)	Syphilis, chlamydia, or gonorrhoea positive	295	32/74	24/165	3.0 (1.6–5.4)	<0.001
MacLean (2018)	Malawi	FSWs	Depression (PHQ-9)	HIV positive	200	12/3	126/59	1.9 (0.5–6.9)	0.3
			PTSD (PCL-C)			10/6	128/56	0.7 (0.3–2.1)	1.6
Ortblad (2020)	Uganda	FSWs	Depression (PHQ-9)	HIV positive	711	57/143	136/375	1.1 (0.8–1.6)	0.6
			Suicide ideation			45/93	148/425	1.4 (0.9–2.1)	0.11
	Zambia		Depression (PHQ-9)	HIV positive	682	65/86	158/373	1.8 (1.2–2.6)	0.002
			Suicide ideation			85/142	138/317	1.4 (1.0–1.9)	0.06
Peitzmeier (2014)	Gambia	FSWs	Sad or depressed mood for more than 2 weeks at a time in past 3 years	HIV positive	246	31/123	9/87	2.4 (1.1–5.4)	0.03
Poliah (2017)	South Africa	FSWs	Depression (PHQ-9)	HIV positive	150	93/17	22/6	1.5 (0.5–4.2)	0.5
Shen (2016)	China	FSWs	Depression (GHQ)	HIV positive	653	3/339	1/310	2.7 (0.3–26.5)	0.4
				Syphilis positive		13/329	20/291	0.6 (0.3–1.2)	0.1
				Hepatitis C positive		8/334	5/306	1.5 (0.5–4.5)	0.5

^1^Odds in the exposed (e.g., Depression and Violence/Depression and No Violence).

^2^Odds in the unexposed (e.g., No Depression and Violence/No Depression and No Violence).

*Studies use same data source.

***Studies use same data source but different cut-off for depression.

**Abbreviations:** AUDIT, Alcohol Use Disorders Identification Test; BSI, Brief Symptom Inventory; CES-D, Centre for Epidemiological Studies Depression Scale; FSW, female sex worker; GHQ, General Health Questionnaire; HIV, human immunodeficiency virus; OR, odds ratio; PCL-C, PTSD CheckList Civilian Version; PHQ, Patient Health Questionnaire; PTSD, post-traumatic stress disorder; STI, sexually transmitted infection.

**Table 3:** Mental health problems and associations with common risk factors.

Risk factor	Total number of studies	Number of studies included in meta-analysis	Pooled OR (95% CI)	*P* value
**Violence**				
Depression and violence (recent or ever)	13	7	2.2 (1.4–3.3)	<0.001
Depression and recent violence	6	5	2.3 (1.3–4.2)	0.005
Recent suicide attempt and violence (recent or ever)	3	2	3.5 (2.3–5.5)	<0.001
**Alcohol and drug use**				
Depression and alcohol use	5	4 [3 with outlier removed]	1.6 (0.8–3.1), 2.1 (1.4–3.2)	0.2, <0.001
Recent suicide ideation and alcohol use	2	2	1.6 (1.0–2.5)	0.003
Depression and illicit drug use	3	2	2.1 (1.4–3.1)	<0.001
**Condom use**				
Depression and inconsistent condom use with clients	7	6	1.6 (1.2–2.1)	0.001
Depression and inconsistent condom use with a regular partner	2	2	0.7 (0.3–1.9)	0.5
**HIV and STIs**				
Depression and HIV	4	4 (5 countries)	1.4 (1.1–1.8)	0.005
Suicidal ideation (ever or recent) and HIV	2	2 (3 countries)	1.4 (1.1–1.8)	0.04

**Abbreviations:** HIV, human immunodeficiency virus; STI, sexually transmitted infection

#### Violence

Seventeen studies reported on associations between mental health problems and violence experience [30,34,36,37,39,43,44,46,49,52,53,57,59,75,79,80,82], usually by an intimate partner or a client (Table 2). Measures of violence varied by timeframe (recent versus ever), typology (physical, sexual, emotional) and perpetrator (client, intimate partner, etc.). Overall, 13 studies reported associations between depression and violence [34,37,39,43,44,46,49,52,53,59,79,83], with 7 studies included in the meta-analyses (S1 Fig). The pooled unadjusted OR of depression and violence experience (ever or recent) is 2.2 (1.4–3.3), *p* < 0.001 (*n* = 7 studies), and the pooled unadjusted OR of depression and recent violence experience is 2.3 (1.3–4.2), *p* = 0.005 (*n* = 5 studies). Two studies [43,46] reported associations between PTSD and violence experience (ever or recent), with only one of these eligible for inclusion in a meta-analysis (unadjusted OR is 1.5 [0.8–2.6], *p* = 0.13) [46]. One study [30] with HIV-negative FSWs reported associations between psychological distress and recent violence by clients (unadjusted OR 2.0 [1.6–2.4], *p* < 0.001). One study reported suicide ideation ever and physical (unadjusted OR 1.7 [1.2–2.5], *p* = 0.008) or sexual violence experience ever (unadjusted OR 2.9 [2.0–4.2], *p* < 0.001) [36], and 2 studies reported recent suicidal ideation and violence experience (ever or recent), with only one of these eligible for inclusion in a meta-analysis (unadjusted OR 2.5 [1.3–4.7], *p* = 0.004) [79]. Three studies reported recent suicide attempt and violence experience (ever or recent), with 2 of these eligible for inclusion in a meta-analysis (S1 Fig). The pooled unadjusted OR of recent suicide attempt and violence experience (ever or recent) is 3.5 [2.2–5.5], *p* < 0.001.

Three studies reported on police violence (harassment, arrest, or raids) and mental health problems (Table 2). While no association was found between police harassment (ever) and current depression (unadjusted OR 0.9 [0.4–2.3], *p* = 0.9) [49], police arrest (ever) was associated with current depression in one study by Patel and colleagues (unadjusted OR 2.2 [1.7–2.8], *p* < 0.001) [53], and police raid in the past year was associated with a suicide attempt in the past 3 months (unadjusted OR 2.3 [1.2–4.3], *p* = 0.01) in a study by Shahmanesh and colleagues [80].

#### Alcohol and drug use

Associations between mental health problems and alcohol use were reported by 6 studies, but there was marked variation in how alcohol use was measured, with 2 studies asking about alcohol use in the past 30 days [53] or alcohol intoxication in the past 6 months [56] and 4 studies using Alcohol Use Disorders Identification Test (AUDIT) to measure hazardous, harmful, or dependent drinking [44,73,84] or severe binge drinking [46] (Table 2). The pooled unadjusted OR for depression and alcohol use is 1.6 (0.8–3.1), *p =* 0.2 (*n =* 4 studies) (S2 Fig); when the outlier study is removed from the analyses, the pooled unadjusted OR is 2.1 (1.4*–*3.2), *p* < 0.001 (*n =* 3 studies) (S2 Fig). Psychological distress and harmful drinking was reported by one study (unadjusted OR 1.0 [0.7–1.4], *p =* 1.0) [73]. The pooled unadjusted OR of recent suicide ideation and alcohol use is 1.6 (1.0*–*2.5), *p =* 0.03 (*n =* 2 studies) (S2 Fig); one study reported associations between a recent suicide attempt and alcohol use, with an unadjusted OR of 2.8 (1.4*–*5.5), *p =* 0.003.

Three studies reported on mental health problems and illicit drug use, again with considerable variation in the way illicit drug use was measured (any illicit drug use ever [85,86] versus polydrug use past month [44]). Two studies were included in the meta-analysis (S3 Fig). The pooled unadjusted OR for depression and illicit drug use is 2.1 (1.4*–*3.1), *p* < 0.001.

#### Condom use

Nine studies reported on mental health problems and condom use with clients and regular partners [24,53,56,60,62,68,71,80,84]. Condom use measurement varied with studies either reporting frequency of condom use (always versus not always) or condom use at last sex (yes/no). The pooled unadjusted OR for depression and inconsistent condom use with clients is 1.6 (1.2–2.1), *p =* 0.001 (*n =* 6 studies) (S3 Fig). The pooled unadjusted OR for depression and inconsistent condom use with a regular partner is 0.7 (0.3–1.9), *p =* 0.5 (*n =* 2 studies) (S3 Fig). One study reported on recent suicide attempt and inconsistent condom use with clients; the unadjusted OR was 4.3 (2.1–8.7), *p* < 0.001.

#### HIV/STIs

Eight studies reported on HIV/STI and mental health problems [36,44,47–49,60,69,73]. One study [48] was excluded from the meta-analyses because it did not use a validated tool to measure depression, and one study was excluded because it only sampled HIV-negative women [44]. The pooled unadjusted OR for depression and HIV is 1.4 (1.1–1.8), *p =* 0.005 (*n =* 4 studies from 5 countries) and for suicidal ideation and HIV is 1.4 (1.1–1.8), *p =* 0.04 (*n =* 2 studies from 3 countries) (S4 Fig). One study reported associations between depression and current syphilis infection; the unadjusted OR was 0.6 (0.3–1.2), *p =* 0.1 [60].

## Discussion

In this systematic review and meta-analysis using data from 56 studies and 24,940 participants, we found that mental health problems are highly prevalent among FSWs in LMICs and are strongly associated with social and behavioural factors commonly experienced by FSWs. Of note, all studies were cross-sectional, and not a single intervention study designed to address mental disorders among FSWs was identified. The prevalence of mental disorders among FSWs in LMICs was much higher compared with the general population in LMICs. For example, data from 41 LMICs from the 2002–2004 World Health Survey found the prevalence of depression to range between 3.9% and 7.8%, with higher rates among women (7.0%–7.8%) compared with men (3.9%–4.9%) [87]. Additionally, the 12-month prevalence of suicidal behaviour among people in LMICs has been reported to be 2% for suicidal ideation and 0.4% for suicide attempts, with rates higher among women compared with men (ideation: 2.4% women versus 1.6% men; attempt: 0.5% women versus 0.4% men) [88]. FSWs face increased levels of key risk factors for mental disorders and suicidal behaviour, including financial stress, low education, inadequate housing, violence, alcohol and drug use, STIs including HIV, and stigma and discrimination [15, 17, 53, 67], which may help explain the higher prevalence of mental health problems in comparison with the general population. Indeed, findings from our meta-analyses support this hypothesis. Understanding how these social determinants interact with mental disorders and which are modifiable within programmatic timeframes will be crucial to designing holistic interventions for FSWs.

This review adhered to PRISMA guidelines and used a comprehensive search strategy, independent screening and quality appraisal of studies. This study had some limitations. By limiting the search to published studies only, and to literature written in English, we may have missed key studies. We used unadjusted ORs to examine associations between mental health problems and key risk factors to allow like-for-like comparisons between studies; not adjusting for potential confounders may have biased the findings although unadjusted and adjusted ORs were usually similar in individual studies. Where individual studies provided multiple estimates on co-linear outcomes (e.g., depression and violence; depression and police arrest), using unadjusted ORs to calculate the individual associations may have led to participants who had not experienced one outcome (e.g., police arrest) but who had experienced the other (e.g., violence) being included in the reference group and subsequent underestimation of the true association. The removal of studies that sampled participants based on characteristics that are known to be an independent risk factor for mental health problems (such as HIV status, harmful alcohol use) led to fewer studies being included and wider confidence intervals around prevalence estimates and pooled ORs. However, when we re-ran the analyses to include all qualifying studies, regardless of sampling criteria, we did indeed find that estimates were slightly higher, suggesting that inclusion of these studies would have led to an overestimation of the pooled estimates and associations. Several methodological issues across the studies were also observed. All studies were cross-sectional. Longitudinal studies are needed to ascertain direction of causality between mental health problems and other factors common to FSWs, although studies with the general population suggest that these relationships are likely to be bidirectional [89]. Most studies used nonprobability sampling across a wide variety of settings which may introduce selection bias and mean that the most vulnerable women will be missed from these surveys. This in turn may lead to underestimations of mental health estimates. A range of measurement tools was used to capture mental health outcomes, as well as violence, alcohol and drug use, and condom use. Even when studies used the same mental health outcome measures, different cut-off scores were applied. This limits the comparability and reliability of findings across studies and points to a need for establishing more rigorous guidelines on using validated tools with this study population.

To our knowledge, this systematic review is the first globally to estimate the prevalence of mental health problems among FSWs in LMICs and to examine associations between poor mental health and other risk factors common in sex workers’ lives. Our findings and meta-analyses suggest that FSWs experience a high burden of depression, anxiety, PTSD, psychological distress, and suicidal behaviours and that poor mental health is strongly associated with violence experience, drug use, inconsistent condom use, and HIV/STI. Together, this supports the concept of overlapping vulnerabilities and has several important implications.

First, there are no existing studies that we are aware of that describe mental health interventions; low-cost, effective interventions for FSWs with mental health disorders are urgently needed. Among the general population attending primary care services in India and elsewhere, brief psychological interventions delivered by trained lay-counsellors have been shown to effectively treat depression [90,91]. Strategies to prevent suicide could include promoting mental health, limiting access to the means for suicide, reducing harmful alcohol use and violence experience, and training “gatekeepers” to support women at increased risk, such as those who have previously attempted suicide [6]. Such interventions should also be suitable for FSWs and could be adapted and embedded within existing HIV service provision. Second, the strong associations between mental health disorders and key occupational risk factors such as violence and harmful alcohol and drug use support the need for upstream structural interventions as part of holistic HIV prevention programming for FSWs. Again, violence interventions have been shown to be effective in reducing violence among women in LMICs [92,93] as well as among FSWs [94]. Low-cost, brief psychological interventions to treat harmful alcohol use could also be adapted to FSW settings [95]. Third, strong associations between poor mental health and reduced condom use with clients and with HIV infection suggest that treatment of mental health problems may also improve condom use with clients and the sexual and reproductive health of FSWs. In addition, women diagnosed with HIV may require on-going counselling and support, for example, by HIV testing and screening counsellors or FSW peer advocates, which goes beyond CD4 counts and treatment adherence, to also enquire about a woman’s ongoing psychological well-being.

## Supporting information

S1 PRISMA Checklist PRISMA, Preferred Reporting Items for Systematic reviews and Meta-Analyses. (DOC)

S1 Text Database search strategies. (DOCX)

S2 Text CEBM critical appraisal tool. CEBM, Centre for Evidence-Based Management.
(DOCX)

S3 Text Quality assessment of quantiative studies. (DOCX)

S1 Fig Meta-analyses summarising the associations between mental health problems and violence. (TIF)

S2 Fig Meta-analyses summarising the associations between mental health problems and alcohol use. (TIF)

S3 Fig Meta-analyses summarising the associations between mental health problems and illicit drug use and condom use with clients and intimate partners. (TIF)

S4 Fig Meta-analyses summarising the associations between mental health problems and HIV infection. HIV, human immunodeficiency virus.
(TIF)

## Abbreviations

AUDIT: Alcohol Use Disorders Identification Test
CEBM: Centre for Evidence-Based Management
FSW: female sex worker
HIV: human immunodeficiency virus
IQR: interquartile range
LMIC: low- and middle-income country
OR: odds ratio
PRISMA: Preferred Reporting Items for Systematic reviews and Meta-Analysis
PTSD: post-traumatic stress disorder
STI: sexually transmitted infection
UNAIDS: The Joint United Nations Programme on HIV/AIDS
