# Supplementary material for: Mental health problems among female sex workers in low- and middle-income countries: A systematic review and meta-analysis
Source: PLoS Med. 2020 Sep 15;17(9):e1003297. doi: 10.1371/journal.pmed.1003297 (PMC7491736; doi:10.1371/journal.pmed.1003297)
Supplement: S1 Text — (DOCX) [file pmed.1003297.s002.docx]

**Search Strategy for Ovid (EMBASE, PsycINFO, Global Health, Ovid MEDLINE)**

| **#** | **Searches** |
| --- | --- |
| 1 | (mental health or mental well-being or mental wellbeing or psycholog* symptom* or psycholog* health or psycholog* distress or distress or mental illness* or mental disorder* or mental health problem* or mental health issue* or psychiatr* problem* or psychiatr* symptom* or psychiatr* comorbidit* or psychiatry* morbidit* or anxiety or anxiety disorder* or depress* or mood disorder* or depress* symptom* or suicid* or trauma or post-traumatic stress disorder or PTSD).ab,ti. |
| 2 | (sex work* or female sex work* or prostitut* or female prostitut* or sex trad* or transact* sex or FSW* or commercial sex or sex-trade worker*).ab,ti. |
| 3 | (Afghanistan or Albania or Algeria or Angola or Antigua or Barbuda or Argentina or Armenia or Armenian or Aruba or Azerbaijan or Bahrain or Bangladesh or Barbados or Benin or Byelarus or Byelorussian or Belarus or Belorussian or Belorussia or Belize or Bhutan or Bolivia or Bosnia or  Herzegovina or Hercegovina or Botswana or Brasil or Brazil or Bulgaria or Burkina Faso or Burkina Fasso or Upper Volta or Burundi or Urundi or Cambodia or Khmer Republic or Kampuchea or Cameroon or Cameroons or Cameron or Camerons or Cape Verde or Central African Republic or Chad or Chile or China or Colombia or Comoros or Comoro Islands or Comores or Mayotte or Congo or Zaire or Costa Rica or Cote d'Ivoire or Ivory Coast or Croatia or Cuba or Cyprus or Czechoslovakia or Czech Republic or Slovakia or Slovak Republic or Djibouti or French Somaliland or Dominica or Dominican Republic or East Timor or East Timur or Timor Leste or Ecuador or Egypt or United Arab Republic or El Salvador or Eritrea or Estonia or Ethiopia or Fiji or Gabon or Gabonese Republic or Gambia or Gaza or Georgia Republic or Georgian Republic or Ghana or Gold Coast or Greece or Grenada or Guatemala or Guinea or Guam or Guiana or Guyana or Haiti or Honduras or Hungary or India or Maldives or Indonesia or Iran or Iraq or Isle of Man or Jamaica or Jordan or Kazakhstan or Kazakh or Kenya or Kiribati or Korea or Kosovo or Kyrgyzstan or Kirghizia or Kyrgyz Republic or Kirghiz or Kirgizstan or Lao PDR or Laos or Latvia or Lebanon or Lesotho or Basutoland or Liberia or Libya or Lithuania or Macedonia or Madagascar or Malagasy Republic or Malaysia or Malaya or Malay or Sabah or Sarawak or Malawi or Nyasaland or Mali or Malta or Marshall Islands or Mauritania or Mauritius or Agalega Islands or Mexico or Micronesia or Middle East or Moldova or Moldovia or Moldovian or Mongolia or Montenegro or Morocco or Ifni or Mozambique or Myanmar or Myanma or Burma or Namibia or Nepal or Netherlands Antilles or New Caledonia or Nicaragua or Niger or Nigeria  or Northern Mariana Islands or Oman or Muscat or Pakistan or Palau or Palestine or Panama or Paraguay or Peru or Philippines or Philipines or Phillipines or Phillippines or Poland or Portugal or Romania or Rumania or Roumania or Russia or Russian or Rwanda or Ruanda or Saint Kitts or St Kitts or Nevis or Saint Lucia or St Lucia or Saint Vincent or St Vincent or Grenadines or Samoa or Samoan Islands or Navigator Island or Navigator Islands or Sao Tome or Saudi Arabia or Senegal or Serbia or Montenegro or Seychelles or Sierra Leone or Slovenia or Sri Lanka or Ceylon or Solomon Islands or Somalia or South Africa or Sudan or Suriname or Surinam or Swaziland or Syria or Tajikistan or Tadzhikistan or Tadjikistan or Tadzhik or Tanzania or Thailand or Togo or Togolese Republic or Tonga or Trinidad or Tobago or Tunisia or Turkey or Turkmenistan or Turkmen or Uganda or Ukraine or Uruguay or USSR or Soviet Union or Union of Soviet Socialist Republics or Uzbekistan or Uzbek or Vanuatu or New Hebrides or Venezuela or Vietnam or Viet Nam or West Bank or Yemen or Yugoslavia or Zambia or Zimbabwe or Rhodesia or (low and middle income countr*) or low-income countr* or low income countr or middle-income countr* or middle income countr* or LAMIC* or LMIC* or Africa or Central Africa  or Latin America or Caribbean or West Indies or Eastern Europe or Soviet or South America or Arab or Middle East or Latin America or Central America or developing or less developed or under developed or underserved or deprived or poor countr* or transition* countr*).ab,ti. |
| 4 | 1 and 2 and 3 |

**Search Strategy for Web of Science**

| **#** | **Searches** |
| --- | --- |
| #1 | TS=("mental health" OR "mental well-being" OR "mental wellbeing" OR "psycholog* symptom*" OR "psycholog* health" OR "psycholog* distress" OR "distress" OR "mental distress" OR "mental illness*" OR "mental disorder*" OR "mental health problem*" OR "mental health issue*" OR "psychiatr* problem*" OR "psychiatr* symptom*" OR "psychiatr* comorbidit*" OR “psychiatr* morbidit*" OR "anxiety" OR "anxiety disorder*" OR "depress*" OR “depressed mood” OR "mood disorder*" OR "depress* symptom*" OR "suicid*" OR "trauma" OR "post-traumatic stress disorder" OR "PTSD")  *DocType=All document types; Language=All languages;* |
| #2 | TS=("sex work*" OR "female sex work*" OR "prostitut*" OR "female prostitut*" OR "sex trad*" OR "transact* sex" OR "FSW*" OR "commercial sex" OR "sex-trade worker*”)  *DocType=All document types; Language=All languages;* |
| #3 | TS=(“Afghanistan” OR “Albania” OR “Algeria” OR “Angola” OR “Antigua” OR “Barbuda” OR “Argentina” OR “Armenia” OR “Armenian” OR “Aruba” OR “Azerbaijan” OR “Bahrain” OR “Bangladesh” OR “Barbados” OR “Benin” OR “Byelarus” OR “Byelorussian” OR “Belarus” OR “Belorussian” OR “Belorussia” OR “Belize” OR “Bhutan” OR “Bolivia” OR “Bosnia” OR “Herzegovina” OR “Hercegovina” OR “Botswana” OR “Brasil” OR “Brazil” OR “Bulgaria” OR “Burkina Faso” OR “Burkina Fasso” OR “Upper Volta” OR “Burundi” OR “Urundi” OR “Cambodia” OR “Khmer Republic” OR “Kampuchea” OR “Cameroon” OR “Cameroons” OR “Cameron” OR “Camerons” OR “Cape Verde” OR “Central African Republic” OR “Chad” OR “Chile” OR “China” OR “Colombia” OR “Comoros” OR “Comoro Islands” OR “Comores” OR “Mayotte” OR “Congo” OR “Zaire” OR “Costa Rica” OR “Cote d'Ivoire” OR “Ivory Coast” OR “Croatia” OR “Cuba” OR “Cyprus” OR “Czechoslovakia” OR “Czech Republic” OR “Slovakia” OR “Slovak Republic” OR “Djibouti” OR “French Somaliland” OR “Dominica” OR “Dominican Republic” OR “East Timor” OR “East Timur” OR “Timor Leste” OR “Ecuador” OR “Egypt” OR “United Arab Republic” OR “El Salvador” OR “Eritrea” OR “Estonia” OR “Ethiopia” OR “Fiji” OR “Gabon” OR “Gabonese Republic” OR “Gambia” OR “Gaza” OR “Georgia Republic” OR “Georgian Republic” OR “Ghana” OR “Gold Coast” OR “Greece” OR “Grenada” OR “Guatemala” OR “Guinea” OR “Guam” OR “Guiana” OR “Guyana” OR “Haiti” OR “Honduras” OR “Hungary” OR “India” OR “Maldives” OR “Indonesia” OR “Iran” OR “Iraq” OR “Isle of Man” OR “Jamaica” OR “Jordan” OR “Kazakhstan” OR “Kazakh” OR “Kenya” OR “Kiribati” OR “Korea” OR “Kosovo” OR “Kyrgyzstan” OR “Kirghizia” OR “Kyrgyz Republic” OR “Kirghiz” OR “Kirgizstan” OR “Lao PDR” OR “Laos” OR “Latvia” OR “Lebanon” OR “Lesotho” OR “Basutoland” OR “Liberia” OR “Libya” OR “Lithuania” OR “Macedonia” OR “Madagascar” OR “Malagasy Republic” OR “Malaysia” OR “Malaya” OR “Malay” OR “Sabah” OR “Sarawak” OR “Malawi” OR “Nyasaland” OR “Mali” OR “Malta” OR “Marshall Islands” OR “Mauritania” OR “Mauritius” OR “Agalega Islands” OR “Mexico” OR “Micronesia” OR “Middle East” OR “Moldova” OR “Moldovia” OR “Moldovian” OR “Mongolia” OR “Montenegro” OR “Morocco” OR “Ifni” OR “Mozambique” OR “Myanmar” OR “Myanma” OR “Burma” OR “Namibia” OR “Nepal” OR “Netherlands Antilles” OR “New Caledonia” OR “Nicaragua” OR “Niger” OR “Nigeria” OR “Northern Mariana Islands” OR “Oman” OR “Muscat” OR “Pakistan” OR “Palau” OR “Palestine” OR “Panama” OR “Paraguay” OR “Peru” OR “Philippines” OR “Philipines” OR “Phillipines” OR “Phillippines” OR “Poland” OR “Portugal” OR “Romania” OR “Rumania” OR “Roumania” OR “Russia” OR “Russian” OR “Rwanda” OR “Ruanda” OR “Saint Kitts” OR “St Kitts” OR “Nevis” OR “Saint Lucia” OR “St Lucia” OR “Saint Vincent” OR “St Vincent” OR “Grenadines” OR “Samoa” OR “Samoan Islands” OR “Navigator Island” OR “Navigator Islands” OR “Sao Tome” OR “Saudi Arabia” OR “Senegal” OR “Serbia” OR “Montenegro” OR “Seychelles” OR “Sierra Leone” OR “Slovenia” OR “Sri Lanka” OR “Ceylon” OR “Solomon Islands” OR “Somalia” OR “South Africa” OR “Sudan” OR “Suriname” OR “Surinam” OR “Swaziland” OR “Syria” OR “Tajikistan” OR “Tadzhikistan” OR “Tadjikistan” OR “Tadzhik” OR “Tanzania” OR “Thailand” OR “Togo” OR “Togolese Republic” OR “Tonga” OR “Trinidad” OR “Tobago” OR “Tunisia” OR “Turkey” OR “Turkmenistan” OR “Turkmen” OR “Uganda” OR “Ukraine” OR “Uruguay” OR “USSR” OR “Soviet Union” OR “Union of Soviet Socialist Republics” OR “Uzbekistan” OR “Uzbek” OR “Vanuatu” OR “New Hebrides” OR “Venezuela” OR “Vietnam” OR “Viet Nam” OR “West Bank” OR “Yemen” OR “Yugoslavia” OR “Zambia” OR “Zimbabwe” OR “Rhodesia” OR "low and middle income countr*" OR "low-income countr*" OR "low income countr" OR "middle-income countr*" OR "middle income countr*" OR "LAMIC*" OR "LMIC*" OR “Africa” OR “Central Africa” OR “Latin America” OR Caribbean OR “West Indies” OR “Eastern Europe” OR “Soviet” OR “South America” OR “Arab” OR “Middle East” OR “Latin America” OR “Central America” OR “developing countr*” OR “less developed countr*” OR “under developed countr*” OR “underserved countr*” OR “deprived countr*” OR “poor countr*” OR “transition* countr*”)  *DocType=All document types; Language=All languages;* |
| 4 | #3 AND #2 AND #1  *DocType=All document types; Language=All languages* |

**Search Strategy for PubMed**

| **#** | **Searches** |
| --- | --- |
| 1 | ("mental health"[Text Word] OR "mental well-being"[Text Word] OR "mental wellbeing"[Text Word] OR "distress"[Text Word] OR "mental distress"[Text Word] OR "mental illness*"[Text Word] OR "mental disorder*"[Text Word] OR "mental health problem*"[Text Word] OR "mental health issue*"[Text Word] OR "anxiety"[Text Word] OR "anxiety disorder*"[Text Word] OR "depress*"[Text Word] OR "depressed mood"[Text Word] OR "mood disorder*"[Text Word] OR "suicid*"[Text Word] OR "trauma"[Text Word] OR "post-traumatic stress disorder"[Text Word] OR "PTSD"[Text Word]) AND "humans"[MeSH Terms]) |
| 2 | ("sex work*"[Text Word] OR "female sex work*"[Text Word] OR "prostitut*"[Text Word] OR "FSW*"[Text Word] OR "commercial sex"[Text Word]) AND "humans"[MeSH Terms]) |
| 3 | ("Afghanistan"[Text Word] OR "Albania"[Text Word] OR "Algeria"[Text Word] OR "Angola"[Text Word] OR "Antigua"[Text Word] OR "Barbuda"[Text Word] OR "Argentina"[Text Word] OR "Armenia"[Text Word] OR "Armenian"[Text Word] OR "Aruba"[Text Word] OR "Azerbaijan"[Text Word] OR "Bahrain"[Text Word] OR "Bangladesh"[Text Word] OR "Barbados"[Text Word] OR "Benin"[Text Word] OR "Byelarus"[Text Word] OR "Byelorussian"[Text Word] OR "Belarus"[Text Word] OR "Belorussian"[Text Word] OR "Belorussia"[Text Word] OR "Belize"[Text Word] OR "Bhutan"[Text Word] OR "Bolivia"[Text Word] OR "Bosnia"[Text Word] OR "Herzegovina"[Text Word] OR "Hercegovina"[Text Word] OR "Botswana"[Text Word] OR "Brasil"[Text Word] OR "Brazil"[Text Word] OR "Bulgaria"[Text Word] OR "Burkina Faso"[Text Word] OR "Burkina Fasso"[Text Word] OR "Upper Volta"[Text Word] OR "Burundi"[Text Word] OR "Urundi"[Text Word] OR "Cambodia"[Text Word] OR "Khmer Republic"[Text Word] OR "Kampuchea"[Text Word] OR "Cameroon"[Text Word] OR "Cameroons"[Text Word] OR "Cameron"[Text Word] OR "Cape Verde"[Text Word] OR "Central African Republic"[Text Word] OR "Chad"[Text Word] OR "Chile"[Text Word] OR "China"[Text Word] OR "Colombia"[Text Word] OR "Comoros"[Text Word] OR "Comoro Islands"[Text Word] OR "Comores"[Text Word] OR "Mayotte"[Text Word] OR "Congo"[Text Word] OR "Zaire"[Text Word] OR "Costa Rica"[Text Word] OR "Cote d'Ivoire"[Text Word] OR "Ivory Coast"[Text Word] OR "Croatia"[Text Word] OR "Cuba"[Text Word] OR "Cyprus"[Text Word] OR "Czechoslovakia"[Text Word] OR "Czech Republic"[Text Word] OR "Slovakia"[Text Word] OR "Slovak Republic"[Text Word] OR "Djibouti"[Text Word] OR "French Somaliland"[Text Word] OR "Dominica"[Text Word] OR "Dominican Republic"[Text Word] OR "East Timor"[Text Word] OR "Timor Leste"[Text Word] OR "Ecuador"[Text Word] OR "Egypt"[Text Word] OR "United Arab Republic"[Text Word] OR "El Salvador"[Text Word] OR "Eritrea"[Text Word] OR "Estonia"[Text Word] OR "Ethiopia"[Text Word] OR "Fiji"[Text Word] OR "Gabon"[Text Word] OR "Gabonese Republic"[Text Word] OR "Gambia"[Text Word] OR "Gaza"[Text Word] OR "Georgia Republic"[Text Word] OR "Georgian Republic"[Text Word] OR "Ghana"[Text Word] OR "Gold Coast"[Text Word] OR "Greece"[Text Word] OR "Grenada"[Text Word] OR "Guatemala"[Text Word] OR "Guinea"[Text Word] OR "Guam"[Text Word] OR "Guiana"[Text Word] OR "Guyana"[Text Word] OR "Haiti"[Text Word] OR "Honduras"[Text Word] OR "Hungary"[Text Word] OR "India"[Text Word] OR "Maldives"[Text Word] OR "Indonesia"[Text Word] OR "Iran"[Text Word] OR "Iraq"[Text Word] OR "Isle of Man"[Text Word] OR "Jamaica"[Text Word] OR "Jordan"[Text Word] OR "Kazakhstan"[Text Word] OR "Kazakh"[Text Word] OR "Kenya"[Text Word] OR "Kiribati"[Text Word] OR "Korea"[Text Word] OR "Kosovo"[Text Word] OR "Kyrgyzstan"[Text Word] OR "Kirghizia"[Text Word] OR "Kyrgyz Republic"[Text Word] OR "Kirghiz"[Text Word] OR "Kirgizstan"[Text Word] OR "Lao PDR"[Text Word] OR "Laos"[Text Word] OR "Latvia"[Text Word] OR "Lebanon"[Text Word] OR "Lesotho"[Text Word] OR "Basutoland"[Text Word] OR "Liberia"[Text Word] OR "Libya"[Text Word] OR "Lithuania"[Text Word] OR "Macedonia"[Text Word] OR "Madagascar"[Text Word] OR "Malagasy Republic"[Text Word] OR "Malaysia"[Text Word] OR "Malaya"[Text Word] OR "Malay"[Text Word] OR "Sabah"[Text Word] OR "Sarawak"[Text Word] OR "Malawi"[Text Word] OR "Nyasaland"[Text Word] OR "Mali"[Text Word] OR "Malta"[Text Word] OR "Marshall Islands"[Text Word] OR "Mauritania"[Text Word] OR "Mauritius"[Text Word] OR "Agalega Islands"[Text Word] OR "Mexico"[Text Word] OR "Micronesia"[Text Word] OR "Middle East"[Text Word] OR "Moldova"[Text Word] OR "Moldovia"[Text Word] OR "Moldovian"[Text Word] OR "Mongolia"[Text Word] OR "Montenegro"[Text Word] OR "Morocco"[Text Word] OR "Ifni"[Text Word] OR "Mozambique"[Text Word] OR "Myanmar"[Text Word] OR "Myanma"[Text Word] OR "Burma"[Text Word] OR "Namibia"[Text Word] OR "Nepal"[Text Word] OR "Netherlands Antilles"[Text Word] OR "New Caledonia"[Text Word] OR "Nicaragua"[Text Word] OR "Niger"[Text Word] OR "Nigeria"[Text Word] OR "Northern Mariana Islands"[Text Word] OR "Oman"[Text Word] OR "Muscat"[Text Word] OR "Pakistan"[Text Word] OR "Palau"[Text Word] OR "Palestine"[Text Word] OR "Panama"[Text Word] OR "Paraguay"[Text Word] OR "Peru"[Text Word] OR "Philippines"[Text Word] OR "Philipines"[Text Word] OR "Phillipines"[Text Word] OR "Phillippines"[Text Word] OR "Poland"[Text Word] OR "Portugal"[Text Word] OR "Romania"[Text Word] OR "Rumania"[Text Word] OR "Roumania"[Text Word] OR "Russia"[Text Word] OR "Russian"[Text Word] OR "Rwanda"[Text Word] OR "Ruanda"[Text Word] OR "Saint Kitts"[Text Word] OR "St Kitts"[Text Word] OR "Nevis"[Text Word] OR "Saint Lucia"[Text Word] OR "St Lucia"[Text Word] OR "Saint Vincent"[Text Word] OR "St Vincent"[Text Word] OR "Grenadines"[Text Word] OR "Samoa"[Text Word] OR "Samoan Islands"[Text Word] OR "Sao Tome"[Text Word] OR "Saudi Arabia"[Text Word] OR "Senegal"[Text Word] OR "Serbia"[Text Word] OR "Montenegro"[Text Word] OR "Seychelles"[Text Word] OR "Sierra Leone"[Text Word] OR "Slovenia"[Text Word] OR "Sri Lanka"[Text Word] OR "Ceylon"[Text Word] OR "Solomon Islands"[Text Word] OR "Somalia"[Text Word] OR "South Africa"[Text Word] OR "Sudan"[Text Word] OR "Suriname"[Text Word] OR "Surinam"[Text Word] OR "Swaziland"[Text Word] OR "Syria"[Text Word] OR "Tajikistan"[Text Word] OR "Tadzhikistan"[Text Word] OR "Tadjikistan"[Text Word] OR "Tadzhik"[Text Word] OR "Tanzania"[Text Word] OR "Thailand"[Text Word] OR "Togo"[Text Word] OR "Togolese Republic"[Text Word] OR "Tonga"[Text Word] OR "Trinidad"[Text Word] OR "Tobago"[Text Word] OR "Tunisia"[Text Word] OR "Turkey"[Text Word] OR "Turkmenistan"[Text Word] OR "Turkmen"[Text Word] OR "Uganda"[Text Word] OR "Ukraine"[Text Word] OR "Uruguay"[Text Word] OR "USSR"[Text Word] OR "Soviet Union"[Text Word] OR "Union of Soviet Socialist Republics"[Text Word] OR "Uzbekistan"[Text Word] OR "Uzbek"[Text Word] OR "Vanuatu"[Text Word] OR "New Hebrides"[Text Word] OR "Venezuela"[Text Word] OR "Vietnam"[Text Word] OR "Viet Nam"[Text Word] OR "West Bank"[Text Word] OR "Yemen"[Text Word] OR "Yugoslavia"[Text Word] OR "Zambia"[Text Word] OR "Zimbabwe"[Text Word] OR "Rhodesia"[Text Word] OR "LAMIC*"[Text Word] OR "LMIC*"[Text Word] OR Africa[Text Word] OR "Central Africa"[Text Word] OR "Latin America"[Text Word] OR Caribbean[Text Word] OR "West Indies"[Text Word] OR "Eastern Europe"[Text Word] OR "Soviet"[Text Word] OR "South America"[Text Word] OR Arab[Text Word] OR "Middle East"[Text Word] OR "Latin America"[Text Word] OR "Central America"[Text Word] OR "developing countr*"[Text Word] OR “less developed countr*”[Text Word] OR “under developed countr*”[Text Word] OR “underserved countr*”[Text Word] OR “deprived countr*”[Text Word] OR “poor countr*”[Text Word] OR “transition* countr*”[Text Word]) |
| 4 | 1 AND 2 AND 3 |
