# Supplementary material for: Mental health problems among female sex workers in low- and middle-income countries: A systematic review and meta-analysis
Source: PLoS Med. 2020 Sep 15;17(9):e1003297. doi: 10.1371/journal.pmed.1003297 (PMC7491736; doi:10.1371/journal.pmed.1003297)
Supplement: S2 Text — CEBM, Centre for Evidence-Based Management. (DOCX) [file pmed.1003297.s003.docx]

**CEBM Critical Appraisal for a Survey Tool**

| Appraisal questions | Yes | Can’t tell | No |
| --- | --- | --- | --- |
| *1. Did the study address a clearly focused question / issue?* |  |  |  |
| *2. Is the research method (study design) appropriate for answering the research question?* |  |  |  |
| *3. Is the method of selection of the subjects (employees, teams, divisions, organizations) clearly described?* |  |  |  |
| *4. Could the way the sample was obtained introduce (selection)bias?* |  |  |  |
| *5. Was the sample of subjects representative with regard to the population to which the findings will be referred?* |  |  |  |
| *6. Was the sample size based on pre-study considerations of statistical power?* |  |  |  |
| *7. Was a satisfactory response rate achieved?* |  |  |  |
| *8. Are the measurements (questionnaires) likely to be valid and reliable?* |  |  |  |
| *9. Was the statistical significance assessed?* |  |  |  |
| *10. Are confidence intervals given for the main results?* |  |  |  |
| *11. Could there be confounding factors that haven’t been accounted for?* |  |  |  |
| *12. Can the results be applied to your organization?* |  |  |  |

Adapted from Crombie, *The Pocket Guide to Critical Appraisal*; the critical appraisal approach used by the Oxford Centre for Evidence Medicine, checklists of the Dutch Cochrane Centre, BMJ editor’s checklists and the checklists of the EPPI Centre.
