# Supplementary material for: Mental health problems among female sex workers in low- and middle-income countries: A systematic review and meta-analysis
Source: PLoS Med. 2020 Sep 15;17(9):e1003297. doi: 10.1371/journal.pmed.1003297 (PMC7491736; doi:10.1371/journal.pmed.1003297)
Supplement: S3 Text — (DOCX) [file pmed.1003297.s004.docx]

**Studies and CEMBa scores**

| Author & Study | Country | Q1 | Q2 | Q3 | Q4 | Q5 | Q6 | Q7 | Q8 | Q9 | Q10 | Q11 | Total Score |
| --- | --- | --- | --- | --- | --- | --- | --- | --- | --- | --- | --- | --- | --- |
| **AFRICA** | | | | | | | | | | | | | |
| Abelson (2019) | Cameroon | 1 | 1 | 1 | 0 | 0 | 0 | 1 | 1 | 1 | 1 | 0 | 7 |
| Akinnawo (1995) | Nigeria | 1 | 1 | 0 | 0 | 0 | 0 | 0 | 1 | 1 | 1 | 0 | 5 |
| Barnhart (2019) | Tanzania | 1 | 1 | 1 | 0 | 0 | 0 | 1 | 1 | 1 | 0 | 0 | 6 |
| Berger (2018) | Swaziland | 1 | 1 | 0 | 0 | 0 | 1 | 0 | 0 | 1 | 1 | 0 | 5 |
| Bitty-Anderson (2019) | Togo | 1 | 1 | 1 | 0 | 0 | 0 | 0 | 1 | 1 | 1 | 0 | 6 |
| Cange (2019) | Burkina Faso | 1 | 1 | 0 | 0 | 0 | 0 | 0 | 1 | 1 | 1 | 0 | 5 |
| Coetzee (2018) | South Africa | 1 | 1 | 1 | 0 | 0 | 0 | 1 | 1 | 1 | 1 | 0 | 7 |
| Grosso (2019) | Togo and Burkina Faso | 1 | 1 | 1 | 0 | 0 | 0 | 0 | 0 | 1 | 1 | 0 | 5 |
| Kim (2018) | Burkina Faso | 1 | 1 | 0 | 0 | 0 | 0 | 0 | 1 | 1 | 1 | 0 | 5 |
| Lion (2017) | South Africa | 1 | 1 | 1 | 0 | 0 | 0 | 0 | 1 | 1 | 1 | 0 | 6 |
| MacLean (2018) | Malawi | 1 | 1 | 0 | 0 | 0 | 1 | 1 | 1 | 1 | 1 | 0 | 7 |
| Ortblad (2020) | Uganda, Zambia | 1 | 1 | 1 | 1 | 1 | 1 | 1 | 1 | 1 | 1 | 0 | 10 |
| Peitzmier (2014) | The Gambia | 1 | 1 | 1 | 0 | 0 | 1 | 0 | 0 | 1 | 1 | 0 | 6 |
| Poliah (2017) | South Africa | 1 | 1 | 1 | 0 | 0 | 0 | 0 | 1 | 1 | 0 | 0 | 5 |
| Rhead (2018) | Zimbabwe | 1 | 1 | 1 | 0 | 0 | 0 | 0 | 1 | 1 | 1 | 0 | 6 |
| Roberts (2018) | Kenya | 1 | 1 | 1 | 0 | 0 | 0 | 0 | 1 | 1 | 1 | 0 | 6 |
| **EASTERN MEDITERRANEAN** | | | | | | | | | | | | | |
| Lari (2014) | Iran | 1 | 1 | 1 | 0 | 0 | 0 | 0 | 1 | 0 | 0 | 0 | 4 |
| Ranjbar (2019) | Iran | 1 | 1 | 1 | 0 | 0 | 0 | 0 | 1 | 0 | 0 | 0 | 4 |
| **EUROPE** | | | | | | | | | | | | | |
| Lang (2011) | Armenia | 1 | 1 | 1 | 0 | 0 | 0 | 0 | 1 | 1 | 0 | 0 | 5 |
| **SOUTH EAST ASIA** | | | | | | | | | | | | | |
| Ghose (2015) | India | 1 | 1 | 1 | 0 | 0 | 0 | 1 | 1 | 0 | 0 | 0 | 5 |
| Hengartner (2015) | Bangladesh | 1 | 1 | 1 | 0 | 0 | 0 | 1 | 1 | 1 | 0 | 0 | 6 |
| Iaisuklang (2017) | India | 1 | 1 | 1 | 0 | 0 | 0 | 0 | 1 | 0 | 0 | 0 | 4 |
| Pandiyan (2012) | India | 1 | 0 | 1 | 0 | 0 | 0 | 0 | 1 | 0 | 0 | 0 | 3 |
| Patel (2016) | India | 1 | 1 | 1 | 1 | 1 | 1 | 0 | 1 | 1 | 1 | 0 | 9 |
| Patel (2015) | India | 1 | 1 | 1 | 1 | 1 | 1 | 0 | 1 | 1 | 1 | 0 | 9 |
| Shahmanesh (2009) | India | 1 | 1 | 1 | 0 | 1 | 0 | 0 | 1 | 1 | 1 | 0 | 7 |
| Suresh (2009) | India | 1 | 1 | 0 | 0 | 0 | 0 | 0 | 1 | 1 | 0 | 0 | 4 |
| **WESTERN PACIFIC** | | | | | | | | | | | | | |
| Brody (2016) | Cambodia | 1 | 1 | 0 | 1 | 1 | 1 | 0 | 1 | 1 | 1 | 0 | 8 |
| Carlson (2017) | Mongolia | 1 | 1 | 1 | 0 | 0 | 0 | 0 | 1 | 1 | 0 | 0 | 5 |
| Chen (2017) | China | 1 | 1 | 1 | 0 | 0 | 0 | 0 | 1 | 1 | 0 | 0 | 5 |
| Gu (2010a) | China | 1 | 1 | 1 | 0 | 0 | 0 | 0 | 1 | 1 | 1 | 0 | 6 |
| Gu (2010b) | China | 1 | 1 | 1 | 0 | 0 | 0 | 0 | 0 | 1 | 1 | 0 | 5 |
| Gu (2014) | China | 1 | 1 | 1 | 0 | 0 | 0 | 0 | 1 | 1 | 1 | 0 | 6 |
| Hong (2010) | China | 1 | 1 | 0 | 0 | 0 | 0 | 1 | 1 | 1 | 1 | 0 | 6 |
| Hong (2007a) | China | 1 | 1 | 1 | 0 | 0 | 1 | 0 | 1 | 1 | 1 | 0 | 7 |
| Hong (2007b) | China | 1 | 1 | 1 | 0 | 0 | 0 | 0 | 1 | 1 | 1 | 0 | 6 |
| Hong (2013) | China | 1 | 1 | 1 | 0 | 0 | 1 | 1 | 1 | 1 | 1 | 0 | 8 |
| Huang (2014) | China | 1 | 1 | 1 | 0 | 0 | 0 | 1 | 1 | 1 | 1 | 0 | 7 |
| Jackson (2013) | China | 1 | 0 | 0 | 0 | 0 | 0 | 0 | 1 | 1 | 1 | 0 | 4 |
| Muth (2017) | Cambodia | 1 | 1 | 0 | 0 | 0 | 1 | 0 | 1 | 1 | 1 | 0 | 6 |
| Offringa (2017) | Mongolia | 1 | 1 | 1 | 1 | 1 | 0 | 0 | 1 | 1 | 0 | 0 | 7 |
| Sagtani (2013) | Nepal | 1 | 1 | 1 | 0 | 0 | 1 | 1 | 1 | 1 | 1 | 0 | 8 |
| Shen (2016) | China | 1 | 1 | 1 | 0 | 0 | 0 | 0 | 1 | 1 | 1 | 0 | 6 |
| Shrestha (2017) | Nepal | 1 | 1 | 1 | 0 | 0 | 0 | 1 | 1 | 1 | 1 | 0 | 7 |
| Urada (2013) | Philippines | 1 | 1 | 1 | 0 | 0 | 0 | 0 | 1 | 1 | 1 | 0 | 6 |
| Witte (2010) | Mongolia | 1 | 1 | 1 | 0 | 0 | 0 | 0 | 1 | 0 | 0 | 0 | 4 |
| Yang (2005) | China | 1 | 1 | 1 | 0 | 0 | 0 | 0 | 1 | 1 | 1 | 0 | 6 |
| **AMERICAS** | | | | | | | | | | | | | |
| Devóglio (2017) | Brazil | 1 | 1 | 1 | 0 | 0 | 0 | 0 | 1 | 1 | 0 | 0 | 5 |
| González-Forteza (2014) | Mexico | 1 | 1 | 0 | 0 | 0 | 0 | 0 | 1 | 0 | 0 | 0 | 3 |
| Jain (2019) | Mexico | 1 | 1 | 0 | 0 | 0 | 0 | 0 | 1 | 1 | 1 | 0 | 5 |
| Logie (2018) | Jamaica | 1 | 1 | 1 | 0 | 0 | 0 | 0 | 1 | 1 | 1 | 0 | 6 |
| Rael (2017a) | Dominican Republic | 1 | 1 | 1 | 0 | 0 | 0 | 0 | 1 | 1 | 1 | 0 | 6 |
| Semple (2019) | Mexico | 1 | 1 | 1 | 0 | 0 | 0 | 1 | 1 | 1 | 0 | 0 | 6 |
| Ulibarri (2009) | Mexico | 1 | 1 | 1 | 0 | 0 | 0 | 0 | 1 | 1 | 0 | 0 | 5 |
| Ulibarri (2013) | Mexico | 1 | 1 | 1 | 0 | 0 | 0 | 0 | 1 | 1 | 1 | 0 | 6 |
| Ulibarri (2015) | Mexico | 1 | 1 | 1 | 0 | 0 | 0 | 0 | 1 | 1 | 0 | 0 | 5 |
