# Supplementary figures and images for: Mental health problems among female sex workers in low- and middle-income countries: A systematic review and meta-analysis
Source: PLoS Med. 2020 Sep 15;17(9):e1003297. doi: 10.1371/journal.pmed.1003297 (PMC7491736; doi:10.1371/journal.pmed.1003297)

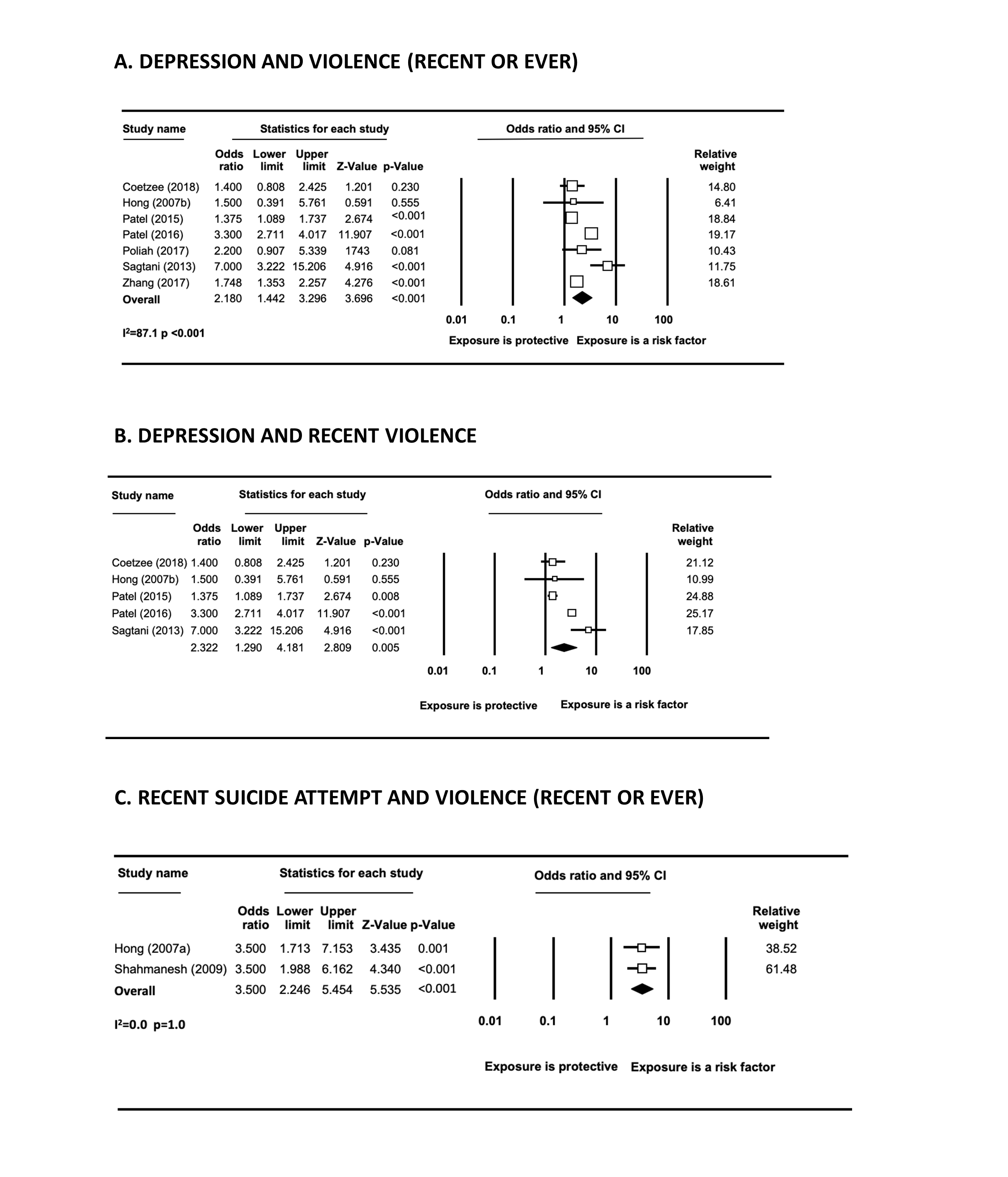

Supplement: S1 Fig — (TIF) [file pmed.1003297.s005.tif]

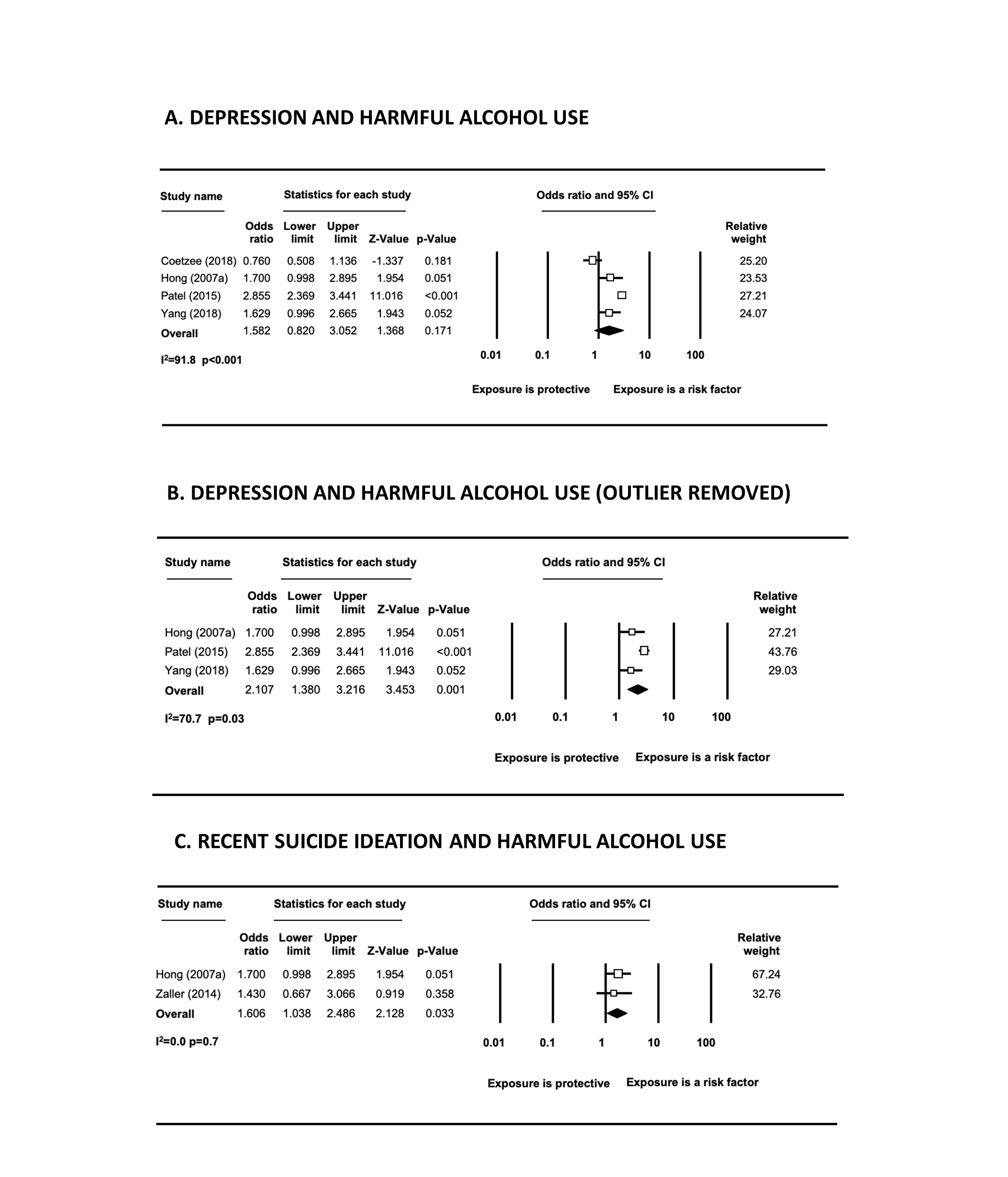

Supplement: S2 Fig — (TIF) [file pmed.1003297.s006.tif]

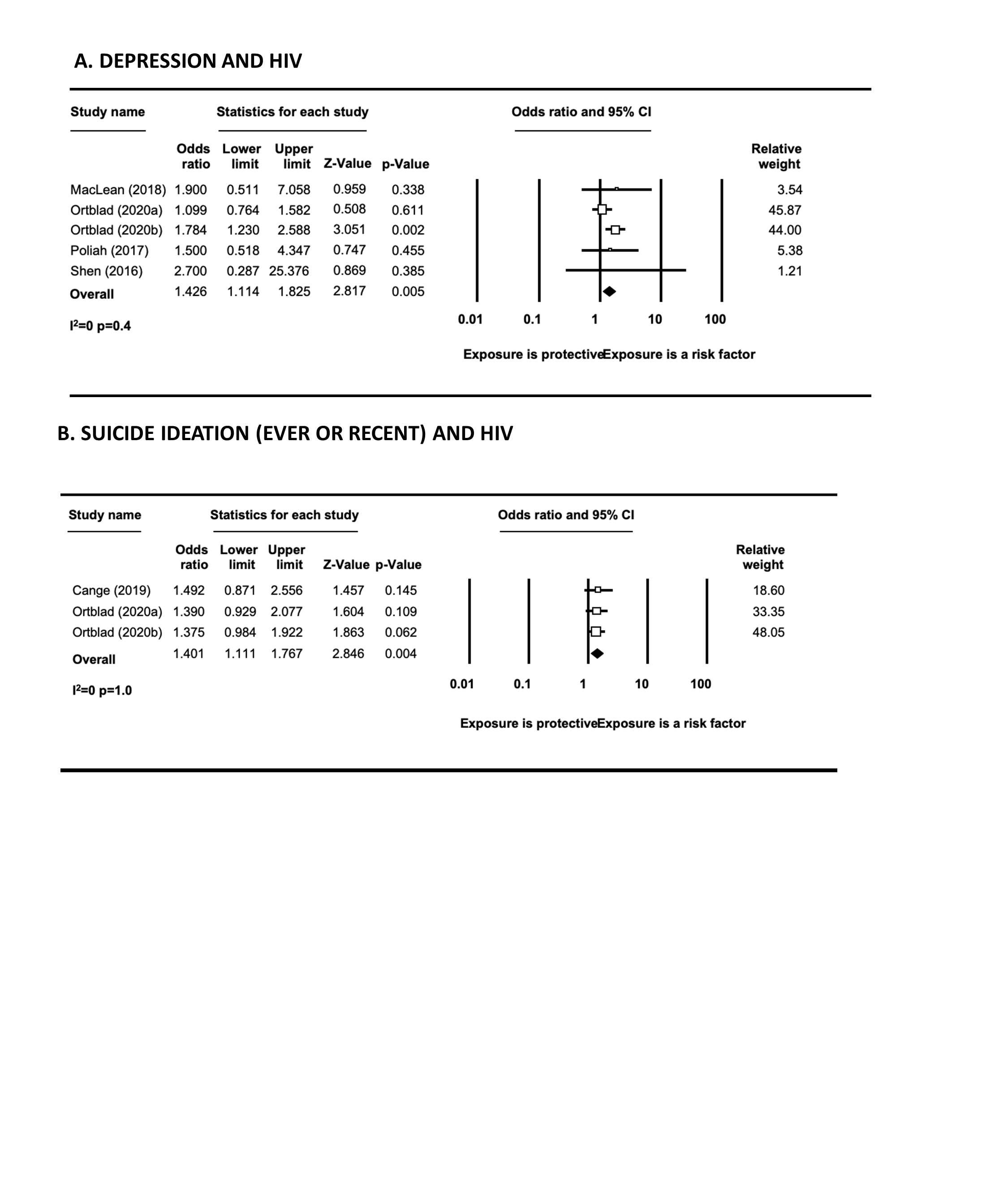

Supplement: S4 Fig — HIV, human immunodeficiency virus. (TIF) [file pmed.1003297.s008.tif]
